# Production of $\mathbf {\Sigma (1385)^{\pm }}$ and $\mathbf {\Xi (1530)^{0}}$ in proton–proton collisions at $\mathbf {\sqrt{s}=}$ 7 TeV

**DOI:** 10.1140/epjc/s10052-014-3191-x

**Published:** 2015-01-10

**Authors:** ALICE Collaboration, B. Abelev, J. Adam, D. Adamová, M. M. Aggarwal, G. Aglieri Rinella, M. Agnello, A. Agostinelli, N. Agrawal, Z. Ahammed, N. Ahmad, I. Ahmed, S. U. Ahn, S. A. Ahn, I. Aimo, S. Aiola, M. Ajaz, A. Akindinov, S. N. Alam, D. Aleksandrov, B. Alessandro, D. Alexandre, A. Alici, A. Alkin, J. Alme, T. Alt, S. Altinpinar, I. Altsybeev, C. Alves Garcia Prado, C. Andrei, A. Andronic, V. Anguelov, J. Anielski, T. Antičić, F. Antinori, P. Antonioli, L. Aphecetche, H. Appelshäuser, S. Arcelli, N. Armesto, R. Arnaldi, T. Aronsson, I. C. Arsene, M. Arslandok, A. Augustinus, R. Averbeck, T. C. Awes, M. D. Azmi, M. Bach, A. Badalà, Y. W. Baek, S. Bagnasco, R. Bailhache, R. Bala, A. Baldisseri, F. Baltasar Dos Santos Pedrosa, R. C. Baral, R. Barbera, F. Barile, G. G. Barnaföldi, L. S. Barnby, V. Barret, J. Bartke, M. Basile, N. Bastid, S. Basu, B. Bathen, G. Batigne, A. Batista Camejo, B. Batyunya, P. C. Batzing, C. Baumann, I. G. Bearden, H. Beck, C. Bedda, N. K. Behera, I. Belikov, F. Bellini, R. Bellwied, E. Belmont-Moreno, R. Belmont, V. Belyaev, G. Bencedi, S. Beole, I. Berceanu, A. Bercuci, Y. Berdnikov, D. Berenyi, M. E. Berger, R. A. Bertens, D. Berzano, L. Betev, A. Bhasin, I. R. Bhat, A. K. Bhati, B. Bhattacharjee, J. Bhom, L. Bianchi, N. Bianchi, C. Bianchin, J. Bielčík, J. Bielčíková, A. Bilandzic, S. Bjelogrlic, F. Blanco, D. Blau, C. Blume, F. Bock, A. Bogdanov, H. Bøggild, M. Bogolyubsky, F. V. Böhmer, L. Boldizsár, M. Bombara, J. Book, H. Borel, A. Borissov, F. Bossú, M. Botje, E. Botta, S. Böttger, P. Braun-Munzinger, M. Bregant, T. Breitner, T. A. Broker, T. A. Browning, M. Broz, E. Bruna, G. E. Bruno, D. Budnikov, H. Buesching, S. Bufalino, P. Buncic, O. Busch, Z. Buthelezi, D. Caffarri, X. Cai, H. Caines, L. Calero Diaz, A. Caliva, E. Calvo Villar, P. Camerini, F. Carena, W. Carena, J. Castillo Castellanos, E. A. R. Casula, V. Catanescu, C. Cavicchioli, C. Ceballos Sanchez, J. Cepila, P. Cerello, B. Chang, S. Chapeland, J. L. Charvet, S. Chattopadhyay, S. Chattopadhyay, V. Chelnokov, M. Cherney, C. Cheshkov, B. Cheynis, V. Chibante Barroso, D. D. Chinellato, P. Chochula, M. Chojnacki, S. Choudhury, P. Christakoglou, C. H. Christensen, P. Christiansen, T. Chujo, S. U. Chung, C. Cicalo, L. Cifarelli, F. Cindolo, J. Cleymans, F. Colamaria, D. Colella, A. Collu, M. Colocci, G. Conesa Balbastre, Z. Conesa del Valle, M. E. Connors, J. G. Contreras, T. M. Cormier, Y. Corrales Morales, P. Cortese, I. Cortés Maldonado, M. R. Cosentino, F. Costa, P. Crochet, R. Cruz Albino, E. Cuautle, L. Cunqueiro, A. Dainese, R. Dang, A. Danu, D. Das, I. Das, K. Das, S. Das, A. Dash, S. Dash, S. De, H. Delagrange, A. Deloff, E. Dénes, G. D’Erasmo, A. De Caro, G. de Cataldo, J. de Cuveland, A. De Falco, D. De Gruttola, N. De Marco, S. De Pasquale, R. de Rooij, M. A. Diaz Corchero, T. Dietel, P. Dillenseger, R. Divià, D. Di Bari, S. Di Liberto, A. Di Mauro, P. Di Nezza, Ø. Djuvsland, A. Dobrin, T. Dobrowolski, D. Domenicis Gimenez, B. Dönigus, O. Dordic, S. Dørheim, A. K. Dubey, A. Dubla, L. Ducroux, P. Dupieux, A. K. Dutta Majumdar, T. E. Hilden, R. J. Ehlers, D. Elia, H. Engel, B. Erazmus, H. A. Erdal, D. Eschweiler, B. Espagnon, M. Esposito, M. Estienne, S. Esumi, D. Evans, S. Evdokimov, D. Fabris, J. Faivre, D. Falchieri, A. Fantoni, M. Fasel, D. Fehlker, L. Feldkamp, D. Felea, A. Feliciello, G. Feofilov, J. Ferencei, A. Fernández Téllez, E. G. Ferreiro, A. Ferretti, A. Festanti, J. Figiel, M. A. S. Figueredo, S. Filchagin, D. Finogeev, F. M. Fionda, E. M. Fiore, E. Floratos, M. Floris, S. Foertsch, P. Foka, S. Fokin, E. Fragiacomo, A. Francescon, U. Frankenfeld, U. Fuchs, C. Furget, A. Furs, M. Fusco Girard, J. J. Gaardhøje, M. Gagliardi, A. M. Gago, M. Gallio, D. R. Gangadharan, P. Ganoti, C. Gao, C. Garabatos, E. Garcia-Solis, C. Gargiulo, I. Garishvili, J. Gerhard, M. Germain, A. Gheata, M. Gheata, B. Ghidini, P. Ghosh, S. K. Ghosh, P. Gianotti, P. Giubellino, E. Gladysz-Dziadus, P. Glässel, A. Gomez Ramirez, P. González-Zamora, S. Gorbunov, L. Görlich, S. Gotovac, L. K. Graczykowski, A. Grelli, A. Grigoras, C. Grigoras, V. Grigoriev, A. Grigoryan, S. Grigoryan, B. Grinyov, N. Grion, J. F. Grosse-Oetringhaus, J.-Y. Grossiord, R. Grosso, F. Guber, R. Guernane, B. Guerzoni, M. Guilbaud, K. Gulbrandsen, H. Gulkanyan, M. Gumbo, T. Gunji, A. Gupta, R. Gupta, K. H. Khan, R. Haake, Ø. Haaland, C. Hadjidakis, M. Haiduc, H. Hamagaki, G. Hamar, L. D. Hanratty, A. Hansen, J. W. Harris, H. Hartmann, A. Harton, D. Hatzifotiadou, S. Hayashi, S. T. Heckel, M. Heide, H. Helstrup, A. Herghelegiu, G. Herrera Corral, B. A. Hess, K. F. Hetland, B. Hippolyte, J. Hladky, P. Hristov, M. Huang, T. J. Humanic, N. Hussain, T. Hussain, D. Hutter, D. S. Hwang, R. Ilkaev, I. Ilkiv, M. Inaba, G. M. Innocenti, C. Ionita, M. Ippolitov, M. Irfan, M. Ivanov, V. Ivanov, A. Jachołkowski, P. M. Jacobs, C. Jahnke, H. J. Jang, M. A. Janik, P. H. S. Y. Jayarathna, C. Jena, S. Jena, R. T. Jimenez Bustamante, P. G. Jones, H. Jung, A. Jusko, V. Kadyshevskiy, P. Kalinak, A. Kalweit, J. Kamin, J. H. Kang, V. Kaplin, S. Kar, A. Karasu Uysal, O. Karavichev, T. Karavicheva, E. Karpechev, U. Kebschull, R. Keidel, D. L. D. Keijdener, M. Keil SVN, M. M. Khan, P. Khan, S. A. Khan, A. Khanzadeev, Y. Kharlov, B. Kileng, B. Kim, D. W. Kim, D. J. Kim, J. S. Kim, M. Kim, M. Kim, S. Kim, T. Kim, S. Kirsch, I. Kisel, S. Kiselev, A. Kisiel, G. Kiss, J. L. Klay, J. Klein, C. Klein-Bösing, A. Kluge, M. L. Knichel, A. G. Knospe, C. Kobdaj, M. Kofarago, M. K. Köhler, T. Kollegger, A. Kolojvari, V. Kondratiev, N. Kondratyeva, A. Konevskikh, V. Kovalenko, M. Kowalski, S. Kox, G. Koyithatta Meethaleveedu, J. Kral, I. Králik, A. Kravčáková, M. Krelina, M. Kretz, M. Krivda, F. Krizek, E. Kryshen, M. Krzewicki, V. Kučera, Y. Kucheriaev, T. Kugathasan, C. Kuhn, P. G. Kuijer, I. Kulakov, J. Kumar, P. Kurashvili, A. Kurepin, A. B. Kurepin, A. Kuryakin, S. Kushpil, M. J. Kweon, Y. Kwon, P. Ladron de Guevara, C. Lagana Fernandes, I. Lakomov, R. Langoy, C. Lara, A. Lardeux, A. Lattuca, S. L. La Pointe, P. La Rocca, R. Lea, L. Leardini, G. R. Lee, I. Legrand, J. Lehnert, R. C. Lemmon, V. Lenti, E. Leogrande, M. Leoncino, I. León Monzón, P. Lévai, S. Li, J. Lien, R. Lietava, S. Lindal, V. Lindenstruth, C. Lippmann, M. A. Lisa, H. M. Ljunggren, D. F. Lodato, P. I. Loenne, V. R. Loggins, V. Loginov, D. Lohner, C. Loizides, X. Lopez, E. López Torres, X.-G. Lu, P. Luettig, M. Lunardon, G. Luparello, R. Ma, A. Maevskaya, M. Mager, D. P. Mahapatra, S. M. Mahmood, A. Maire, R. D. Majka, M. Malaev, I. Maldonado Cervantes, L. Malinina, D. Mal’Kevich, P. Malzacher, A. Mamonov, L. Manceau, V. Manko, F. Manso, V. Manzari, M. Marchisone, J. Mareš, G. V. Margagliotti, A. Margotti, A. Marín, C. Markert, M. Marquard, I. Martashvili, N. A. Martin, P. Martinengo, M. I. Martínez, G. Martínez García, J. Martin Blanco, Y. Martynov, A. Mas, S. Masciocchi, M. Masera, A. Masoni, L. Massacrier, A. Mastroserio, A. Matyja, C. Mayer, J. Mazer, M. A. Mazzoni, F. Meddi, A. Menchaca-Rocha, E. Meninno, J. Mercado Pérez, M. Meres, Y. Miake, K. Mikhaylov, L. Milano, J. Milosevic, A. Mischke, A. N. Mishra, D. Miśkowiec, J. Mitra, C. M. Mitu, J. Mlynarz, N. Mohammadi, B. Mohanty, L. Molnar, L. Montaño Zetina, E. Montes, M. Morando, D. A. Moreira De Godoy, S. Moretto, A. Morreale, A. Morsch, V. Muccifora, E. Mudnic, D. Mühlheim, S. Muhuri, M. Mukherjee, H. Müller, M. G. Munhoz, S. Murray, L. Musa, J. Musinsky, B. K. Nandi, R. Nania, E. Nappi, C. Nattrass, K. Nayak, T. K. Nayak, S. Nazarenko, A. Nedosekin, M. Nicassio, M. Niculescu, J. Niedziela, B. S. Nielsen, S. Nikolaev, S. Nikulin, V. Nikulin, B. S. Nilsen, F. Noferini, P. Nomokonov, G. Nooren, J. Norman, A. Nyanin, J. Nystrand, H. Oeschler, S. Oh, S. K. Oh, A. Okatan, T. Okubo, L. Olah, J. Oleniacz, A. C. Oliveira Da Silva, J. Onderwaater, C. Oppedisano, A. Ortiz Velasquez, A. Oskarsson, J. Otwinowski, K. Oyama, M. Ozdemir, P. Sahoo, Y. Pachmayer, M. Pachr, P. Pagano, G. Paić, C. Pajares, S. K. Pal, A. Palmeri, D. Pant, V. Papikyan, G. S. Pappalardo, P. Pareek, W. J. Park, S. Parmar, A. Passfeld, D. I. Patalakha, V. Paticchio, B. Paul, T. Pawlak, T. Peitzmann, H. Pereira Da Costa, E. Pereira De Oliveira Filho, D. Peresunko, C. E. Pérez Lara, A. Pesci, V. Peskov, Y. Pestov, V. Petráček, M. Petran, M. Petris, M. Petrovici, C. Petta, S. Piano, M. Pikna, P. Pillot, O. Pinazza, L. Pinsky, D. B. Piyarathna, M. Płoskoń, M. Planinic, J. Pluta, S. Pochybova, P. L. M. Podesta-Lerma, M. G. Poghosyan, E. H. O. Pohjoisaho, B. Polichtchouk, N. Poljak, A. Pop, S. Porteboeuf-Houssais, J. Porter, B. Potukuchi, S. K. Prasad, R. Preghenella, F. Prino, C. A. Pruneau, I. Pshenichnov, M. Puccio, G. Puddu, P. Pujahari, V. Punin, J. Putschke, H. Qvigstad, A. Rachevski, S. Raha, S. Rajput, J. Rak, A. Rakotozafindrabe, L. Ramello, R. Raniwala, S. Raniwala, S. S. Räsänen, B. T. Rascanu, D. Rathee, A. W. Rauf, V. Razazi, K. F. Read, J. S. Real, K. Redlich, R. J. Reed, A. Rehman, P. Reichelt, M. Reicher, F. Reidt, R. Renfordt, A. R. Reolon, A. Reshetin, F. Rettig, J.-P. Revol, K. Reygers, V. Riabov, R. A. Ricci, T. Richert, M. Richter, P. Riedler, W. Riegler, F. Riggi, A. Rivetti, E. Rocco, M. Rodríguez Cahuantzi, A. Rodriguez Manso, K. Røed, E. Rogochaya, S. Rohni, D. Rohr, D. Röhrich, R. Romita, F. Ronchetti, L. Ronflette, P. Rosnet, A. Rossi, F. Roukoutakis, A. Roy, C. Roy, P. Roy, A. J. Rubio Montero, R. Rui, R. Russo, E. Ryabinkin, Y. Ryabov, A. Rybicki, S. Sadovsky, K. Šafařík, B. Sahlmuller, R. Sahoo, P. K. Sahu, J. Saini, S. Sakai, C. A. Salgado, J. Salzwedel, S. Sambyal, V. Samsonov, X. Sanchez Castro, F. J. Sánchez Rodríguez, L. Šándor, A. Sandoval, M. Sano, G. Santagati, D. Sarkar, E. Scapparone, F. Scarlassara, R. P. Scharenberg, C. Schiaua, R. Schicker, C. Schmidt, H. R. Schmidt, S. Schuchmann, J. Schukraft, M. Schulc, T. Schuster, Y. Schutz, K. Schwarz, K. Schweda, G. Scioli, E. Scomparin, R. Scott, G. Segato, J. E. Seger, Y. Sekiguchi, I. Selyuzhenkov, K. Senosi, J. Seo, E. Serradilla, A. Sevcenco, A. Shabetai, G. Shabratova, R. Shahoyan, A. Shangaraev, A. Sharma, N. Sharma, S. Sharma, K. Shigaki, K. Shtejer, Y. Sibiriak, S. Siddhanta, T. Siemiarczuk, D. Silvermyr, C. Silvestre, G. Simatovic, R. Singaraju, R. Singh, S. Singha, V. Singhal, B. C. Sinha, T. Sinha, B. Sitar, M. Sitta, T. B. Skaali, K. Skjerdal, M. Slupecki, N. Smirnov, R. J. M. Snellings, C. Søgaard, R. Soltz, J. Song, M. Song, F. Soramel, S. Sorensen, M. Spacek, E. Spiriti, I. Sputowska, M. Spyropoulou-Stassinaki, B. K. Srivastava, J. Stachel, I. Stan, G. Stefanek, M. Steinpreis, E. Stenlund, G. Steyn, J. H. Stiller, D. Stocco, M. Stolpovskiy, P. Strmen, A. A. P. Suaide, T. Sugitate, C. Suire, M. Suleymanov, R. Sultanov, M. Šumbera, T. J. M. Symons, A. Szabo, A. Szanto de Toledo, I. Szarka, A. Szczepankiewicz, M. Szymanski, J. Takahashi, M. A. Tangaro, J. D. Tapia Takaki, A. Tarantola Peloni, A. Tarazona Martinez, M. Tariq, M. G. Tarzila, A. Tauro, G. Tejeda Muñoz, A. Telesca, K. Terasaki, C. Terrevoli, J. Thäder, D. Thomas, R. Tieulent, A. R. Timmins, A. Toia, V. Trubnikov, W. H. Trzaska, T. Tsuji, A. Tumkin, R. Turrisi, T. S. Tveter, K. Ullaland, A. Uras, G. L. Usai, M. Vajzer, M. Vala, L. Valencia Palomo, S. Vallero, P. Vande Vyvre, J. Van Der Maarel, J. W. Van Hoorne, M. van Leeuwen, A. Vargas, M. Vargyas, R. Varma, M. Vasileiou, A. Vasiliev, V. Vechernin, M. Veldhoen, A. Velure, M. Venaruzzo, E. Vercellin, S. Vergara Limón, R. Vernet, M. Verweij, L. Vickovic, G. Viesti, J. Viinikainen, Z. Vilakazi, O. Villalobos Baillie, A. Vinogradov, L. Vinogradov, Y. Vinogradov, T. Virgili, V. Vislavicius, Y. P. Viyogi, A. Vodopyanov, M. A. Völkl, K. Voloshin, S. A. Voloshin, G. Volpe, B. von Haller, I. Vorobyev, D. Vranic, J. Vrláková, B. Vulpescu, A. Vyushin, B. Wagner, J. Wagner, V. Wagner, M. Wang, Y. Wang, D. Watanabe, M. Weber, S. G. Weber, J. P. Wessels, U. Westerhoff, J. Wiechula, J. Wikne, M. Wilde, G. Wilk, J. Wilkinson, M. C. S. Williams, B. Windelband, M. Winn, C. G. Yaldo, Y. Yamaguchi, H. Yang, P. Yang, S. Yang, S. Yano, S. Yasnopolskiy, J. Yi, Z. Yin, I.-K. Yoo, I. Yushmanov, V. Zaccolo, C. Zach, A. Zaman, C. Zampolli, S. Zaporozhets, A. Zarochentsev, P. Závada, N. Zaviyalov, H. Zbroszczyk, I. S. Zgura, M. Zhalov, H. Zhang, X. Zhang, Y. Zhang, C. Zhao, N. Zhigareva, D. Zhou, F. Zhou, Y. Zhou, Zhou Zhuo, H. Zhu, J. Zhu, X. Zhu, A. Zichichi, A. Zimmermann, M. B. Zimmermann, G. Zinovjev, Y. Zoccarato, M. Zyzak

**Affiliations:** 1 A.I. Alikhanyan National Science Laboratory (Yerevan Physics Institute) Foundation, Yerevan, Armenia; 2 Benemérita Universidad Autónoma de Puebla, Puebla, Mexico; 3 Bogolyubov Institute for Theoretical Physics, Kiev, Ukraine; 4 Department of Physics and Centre for Astroparticle Physics and Space Science (CAPSS), Bose Institute, Kolkata, India; 5 Budker Institute for Nuclear Physics, Novosibirsk, Russia; 6 California Polytechnic State University, San Luis Obispo, CA USA; 7 Central China Normal University, Wuhan, China; 8 Centre de Calcul de l’IN2P3, Villeurbanne, France; 9 Centro de Aplicaciones Tecnológicas y Desarrollo Nuclear (CEADEN), Havana, Cuba; 10 Centro de Investigaciones Energéticas Medioambientales y Tecnológicas (CIEMAT), Madrid, Spain; 11 Centro de Investigación y de Estudios Avanzados (CINVESTAV), Mexico City and Mérida, Mexico; 12 Centro Fermi-Museo Storico della Fisica e Centro Studi e Ricerche “Enrico Fermi”, Rome, Italy; 13 Chicago State University, Chicago, USA; 14 Commissariat à l’Energie Atomique, IRFU, Saclay, France; 15 COMSATS Institute of Information Technology (CIIT), Islamabad, Pakistan; 16 Departamento de Física de Partículas and IGFAE, Universidad de Santiago de Compostela, Santiago de Compostela, Spain; 17 Department of Physics and Technology, University of Bergen, Bergen, Norway; 18 Department of Physics, Aligarh Muslim University, Aligarh, India; 19 Department of Physics, Ohio State University, Columbus, OH USA; 20 Department of Physics, Sejong University, Seoul, South Korea; 21 Department of Physics, University of Oslo, Oslo, Norway; 22 Dipartimento di Fisica dell’Università ‘La Sapienza’ and Sezione INFN, Rome, Italy; 23 Dipartimento di Fisica dell’Università and Sezione INFN, Cagliari, Italy; 24 Dipartimento di Fisica dell’Università and Sezione INFN, Trieste, Italy; 25 Dipartimento di Fisica dell’Università and Sezione INFN, Turin, Italy; 26 Dipartimento di Fisica e Astronomia dell’Università and Sezione INFN, Bologna, Italy; 27 Dipartimento di Fisica e Astronomia dell’Università and Sezione INFN, Catania, Italy; 28 Dipartimento di Fisica e Astronomia dell’Università and Sezione INFN, Padua, Italy; 29 Dipartimento di Fisica ‘E.R. Caianiello’ dell’Università and Gruppo Collegato INFN, Salerno, Italy; 30 Dipartimento di Scienze e Innovazione Tecnologica dell’Università del Piemonte Orientale and Gruppo Collegato INFN, Alessandria, Italy; 31 Dipartimento Interateneo di Fisica ‘M. Merlin’ and Sezione INFN, Bari, Italy; 32 Division of Experimental High Energy Physics, University of Lund, Lund, Sweden; 33 Eberhard Karls Universität Tübingen, Tübingen, Germany; 34 European Organization for Nuclear Research (CERN), Geneva, Switzerland; 35 Faculty of Engineering, Bergen University College, Bergen, Norway; 36 Faculty of Mathematics, Physics and Informatics, Comenius University, Bratislava, Slovakia; 37 Faculty of Nuclear Sciences and Physical Engineering, Czech Technical University in Prague, Prague, Czech Republic; 38 Faculty of Science, P.J. Šafárik University, Kosice, Slovakia; 39 Frankfurt Institute for Advanced Studies, Johann Wolfgang Goethe-Universität Frankfurt, Frankfurt, Germany; 40 Gangneung-Wonju National University, Gangneung, South Korea; 41 Department of Physics, Gauhati University, Guwahati, India; 42 Helsinki Institute of Physics (HIP), Helsinki, Finland; 43 Hiroshima University, Hiroshima, Japan; 44 Indian Institute of Technology Bombay (IIT), Mumbai, India; 45 Indian Institute of Technology Indore (IITI), Indore, India; 46 Inha University, Inchon, South Korea; 47 Institut de Physique Nucléaire d’Orsay (IPNO), Université Paris-Sud, CNRS-IN2P3, Orsay, France; 48 Institut für Informatik, Johann Wolfgang Goethe-Universität Frankfurt, Frankfurt, Germany; 49 Institut für Kernphysik, Johann Wolfgang Goethe-Universität Frankfurt, Frankfurt, Germany; 50 Institut für Kernphysik, Westfälische Wilhelms-Universität Münster, Münster, Germany; 51 Institut Pluridisciplinaire Hubert Curien (IPHC), Université de Strasbourg, CNRS-IN2P3, Strasbourg, France; 52 Institute for Nuclear Research, Academy of Sciences, Moscow, Russia; 53 Institute for Subatomic Physics of Utrecht University, Utrecht, The Netherlands; 54 Institute for Theoretical and Experimental Physics, Moscow, Russia; 55 Institute of Experimental Physics, Slovak Academy of Sciences, Kosice, Slovakia; 56 Institute of Physics, Academy of Sciences of the Czech Republic, Prague, Czech Republic; 57 Institute of Physics, Bhubaneswar, India; 58 Institute of Space Science (ISS), Bucharest, Romania; 59 Instituto de Ciencias Nucleares, Universidad Nacional Autónoma de México, Mexico City, Mexico; 60 Instituto de Física, Universidad Nacional Autónoma de México, Mexico City, Mexico; 61 iThemba LABS, National Research Foundation, Somerset West, South Africa; 62 Joint Institute for Nuclear Research (JINR), Dubna, Russia; 63 Konkuk University, Seoul, South Korea; 64 Korea Institute of Science and Technology Information, Taejeon, South Korea; 65 KTO Karatay University, Konya, Turkey; 66 Laboratoire de Physique Corpusculaire (LPC), Clermont Université, Université Blaise Pascal, CNRS-IN2P3, Clermont-Ferrand, France; 67 Laboratoire de Physique Subatomique et de Cosmologie, Université Grenoble-Alpes, CNRS-IN2P3, Grenoble, France; 68 Laboratori Nazionali di Frascati, INFN, Frascati, Italy; 69 Laboratori Nazionali di Legnaro, INFN, Legnaro, Italy; 70 Lawrence Berkeley National Laboratory, Berkeley, CA USA; 71 Lawrence Livermore National Laboratory, Livermore, CA USA; 72 Moscow Engineering Physics Institute, Moscow, Russia; 73 National Centre for Nuclear Studies, Warsaw, Poland; 74 National Institute for Physics and Nuclear Engineering, Bucharest, Romania; 75 National Institute of Science Education and Research, Bhubaneswar, India; 76 Niels Bohr Institute, University of Copenhagen, Copenhagen, Denmark; 77 Nikhef, National Institute for Subatomic Physics, Amsterdam, The Netherlands; 78 Nuclear Physics Group, STFC Daresbury Laboratory, Daresbury, UK; 79 Nuclear Physics Institute, Academy of Sciences of the Czech Republic, Řež u Prahy, Czech Republic; 80 Oak Ridge National Laboratory, Oak Ridge, TN USA; 81 Petersburg Nuclear Physics Institute, Gatchina, Russia; 82 Physics Department, Creighton University, Omaha, NE USA; 83 Physics Department, Panjab University, Chandigarh, India; 84 Physics Department, University of Athens, Athens, Greece; 85 Physics Department, University of Cape Town, Cape Town, South Africa; 86 Physics Department, University of Jammu, Jammu, India; 87 Physics Department, University of Rajasthan, Jaipur, India; 88 Physik Department, Technische Universität München, Munich, Germany; 89 Physikalisches Institut, Ruprecht-Karls-Universität Heidelberg, Heidelberg, Germany; 90 Politecnico di Torino, Turin, Italy; 91 Purdue University, West Lafayette, IN USA; 92 Pusan National University, Pusan, South Korea; 93 Research Division and ExtreMe Matter Institute EMMI, GSI Helmholtzzentrum für Schwerionenforschung, Darmstadt, Germany; 94 Rudjer Bošković Institute, Zagreb, Croatia; 95 Russian Federal Nuclear Center (VNIIEF), Sarov, Russia; 96 Russian Research Centre Kurchatov Institute, Moscow, Russia; 97 Saha Institute of Nuclear Physics, Kolkata, India; 98 School of Physics and Astronomy, University of Birmingham, Birmingham, UK; 99 Sección Física, Departamento de Ciencias, Pontificia Universidad Católica del Perú, Lima, Peru; 100 Sezione INFN, Bari, Italy; 101 Sezione INFN, Bologna, Italy; 102 Sezione INFN, Cagliari, Italy; 103 Sezione INFN, Catania, Italy; 104 Sezione INFN, Padua, Italy; 105 Sezione INFN, Rome, Italy; 106 Sezione INFN, Trieste, Italy; 107 Sezione INFN, Turin, Italy; 108 SSC IHEP of NRC Kurchatov institute, Protvino, Russia; 109 SUBATECH, Ecole des Mines de Nantes, Université de Nantes, CNRS-IN2P3, Nantes, France; 110 Suranaree University of Technology, Nakhon Ratchasima, Thailand; 111 Technical University of Split FESB, Split, Croatia; 112 The Henryk Niewodniczanski Institute of Nuclear Physics, Polish Academy of Sciences, Kracòw, Poland; 113 Physics Department, The University of Texas at Austin, Austin, TX USA; 114 Universidad Autónoma de Sinaloa, Culiacán, Mexico; 115 Universidade de São Paulo (USP), São Paulo, Brazil; 116 Universidade Estadual de Campinas (UNICAMP), Campinas, Brazil; 117 University of Houston, Houston, TX USA; 118 University of Jyväskylä, Jyväskylä, Finland; 119 University of Liverpool, Liverpool, UK; 120 University of Tennessee, Knoxville, TN USA; 121 University of Tokyo, Tokyo, Japan; 122 University of Tsukuba, Tsukuba, Japan; 123 University of Zagreb, Zagreb, Croatia; 124 Université de Lyon, Université Lyon 1, CNRS/IN2P3, IPN-Lyon, Villeurbanne, France; 125 V. Fock Institute for Physics, St. Petersburg State University, St. Petersburg, Russia; 126 Variable Energy Cyclotron Centre, Kolkata, India; 127 Vestfold University College, Tonsberg, Norway; 128 Warsaw University of Technology, Warsaw, Poland; 129 Wayne State University, Detroit, MI USA; 130 Wigner Research Centre for Physics, Hungarian Academy of Sciences, Budapest, Hungary; 131 Yale University, New Haven, CT USA; 132 Yonsei University, Seoul, South Korea; 133 Zentrum für Technologietransfer und Telekommunikation (ZTT), Fachhochschule Worms, Worms, Germany; 134 CERN, 1211 Geneva 23, Switzerland

## Abstract

The production of the strange and double-strange baryon resonances ($\Sigma (1385)^{\pm }$, $\Xi (1530)^{0}$) has been measured at mid-rapidity ($\left| y \right|$ $<0.5$) in proton–proton collisions at $\sqrt{s}$ $=$ 7 TeV with the ALICE detector at the LHC. Transverse momentum spectra for inelastic collisions are compared to QCD-inspired models, which in general underpredict the data. A search for the $\phi (1860)$ pentaquark, decaying in the $\Xi \pi$ channel, has been carried out but no evidence is seen.

## Introduction

The study of strange baryon resonances in proton–proton (pp) collisions contributes to the understanding of hadron production mechanisms and provides a reference for tuning QCD-inspired event generators. The strange-quark content makes these baryons a valuable tool in understanding production mechanisms, since the initial state colliding projectiles contain no strange valence quarks and therefore all strange particles are created in the collision.

In addition, a measurement of resonance production in the pp system serves as a reference for understanding resonance production in heavy-ion collisions, where resonances, due to their lifetime of a few fm/$c$ being comparable to the lifetime of the hadronic phase, are sensitive probes of the dynamical evolution of the fireball. Previous measurements at a collision energy of $\sqrt{s}$ $=$ 0.2 TeV with the STAR detector at the RHIC have shown that the yields of ${\Sigma }$(1385) in Au–Au in comparison to pp collisions indicate the presence of rescattering and regeneration in the time span between chemical and kinetic freezeout [1]. Forthcoming analysis of strange baryon resonances in Pb–Pb collisions by the ALICE collaboration will further explore those effects at higher energy and density of the colliding system. The results for the ${\Sigma }$(1385)$^{\pm }$ and ${\Xi (1530)^{0}}$ baryons in pp collisions will therefore serve as benchmark.

Measurements of differential ($\mathrm{d}^2N/(\mathrm{d}y \mathrm{d}p_{\mathrm{T}})$) and integrated (d$N$/d$y$) yields of the ${\Sigma }$(1385)$^{\pm }$ and ${\Xi (1530)^{0}}$ baryons are presented at mid-rapidity ($\left| y \right|$ $<0.5$) in inelastic (INEL) pp collisions at $\sqrt{s}$ $=$ 7 TeV, collected with the ALICE detector [2] at the LHC. The differential spectra are compared to Monte Carlo (MC) event generators. The mean transverse momentum $\langle p_{\mathrm{T}}\rangle$ is compared to those of other particles measured in pp collisions with the ALICE detector at both $\sqrt{s}$ $=$ 7 TeV and $\sqrt{s}$ $=$ 0.9 TeV, and with the STAR detector at $\sqrt{s}$ $=$ 0.2 TeV.

The $\Xi$(1530) reconstruction channel $\Xi \pi$ is additionally analysed to investigate evidence of the $\phi (1860)$ pentaquark, previously reported by the NA49 experiment [3]. No such signal was observed by other experiments at different energies and with different beams and reactions [4–14].

This article is organized as follows. Section 2 gives a brief description of the main detectors used for this analysis and the experimental conditions. Section 2.1 describes track and topological selections. Signal extraction methods are presented in Sect. 2.2, and the efficiency corrections in Sect. 2.3. The evaluation of systematic uncertainties is discussed in Sect. 2.4. In Sect. 3, the $p_{\mathrm{T}}$ spectra and the integrated yields of the studied particle species are given and compared to model predictions. In Sect. 4 the search for the $\phi (1860)$ pentaquark is discussed. Conclusions are presented in Sect. 5.

**Table 1:** Particles involved in this analysis and their PDG parameters [17]. Antiparticles are not listed for brevity. From [17], ${\Xi (1530)^{0}}$ $\longrightarrow \Xi + \pi$ has a branching ratio of $\sim$ 100 %, then ${\Xi (1530)^{0}}$ $\longrightarrow$ ${\Xi }$ $^{-}+$ ${\uppi ^+}$ has a branching ratio of $\sim$ 66.7 % due to isospin considerations

	Valence quarks	Mass (MeV/$c^{\mathrm 2}$)	Width/*c* $\tau$	Decay channel	Branching ratio (%)
${\Sigma }$(1385)$^{+}$	uus	1382.80 $\pm$ 0.35	(36.0 $\pm$ 0.7) MeV/$c^{\mathrm 2}$	${\Lambda }$ $+$ ${\uppi ^+}$	87.0 $\pm$ 1.5
${\Sigma }$(1385)$^{-}$	dds	1387.2 $\pm$ 0.5	(39.4 $\pm$ 2.1) MeV/$c^{\mathrm 2}$	${\Lambda }$ $+$ ${\uppi ^-}$	87.0 $\pm$ 1.5
${\Xi (1530)^{0}}$	uss	1531.80 $\pm$ 0.32	(9.1 $\pm$ 0.5) MeV/$c^{\mathrm 2}$	${\Xi }$ $^{-}+$ ${\uppi ^+}$	66.7
${\Xi }$ $^{-}$	dss	1321.71 $\pm$ 0.07	4.91 cm	${\Lambda }$ $+$ ${\uppi ^-}$	99.887 $\pm$ 0.035
${\Lambda }$	uds	1115.683 $\pm$ 0.006	7.89 cm	p$+$ ${\uppi ^-}$	63.9 $\pm$ 0.5

**Table 2:** Track selection criteria. *PV* primary vertex, *DCA* ${_r}$ and *DCA* $_{z}$ distances of closest approach in the transverse plane and in the longitudinal direction, respectively

Common selections	
$|\eta |$	$<$0.8
$p_{\mathrm{T}}$	>0.15 GeV$/c$
Number of TPC clusters	>70
$\chi ^{2}$ per cluster	$<$4
Primary track selections	
DCA$_{z}$ to PV	$<$2 cm
DCA$_{r}$ to PV	$<$7 $\sigma _{\mathrm {DCA}}$($p_{\mathrm{T}}$)
Number of SPD clusters	$\ge$1
PID (${\Sigma }$(1385) analysis only)	
$|$(d$E/$d$x)_\mathrm{{measured}}-$(d$E/$d$x)_\mathrm{{expected}}|$	$<$3 $\sigma _\mathrm{{TPC}}$

## Experiment and data analysis

The ALICE detector [2] is designed to study a variety of colliding systems, including pp and lead-lead (Pb–Pb) collisions, at TeV-scale energies. The sub-detectors used in this analysis are described in the following. A six-layer silicon inner tracking system (ITS) [15] and a large-volume time projection chamber (TPC) [16] enable charged particle reconstruction with excellent momentum and spatial resolution in full azimuth down to a $p_{\mathrm{T}}$ of 100 MeV$/c$ in the pseudorapidity range $|\eta |<0.9$. The primary interaction vertex is determined with the TPC and ITS detectors with a resolution of 200 $\upmu$m for events with few tracks ($N_\mathrm{{ch}}\simeq 3$) and below 100 $\upmu$m for events with higher multiplicity ($N_\mathrm{{ch}}\gtrsim 25$). In addition, both detectors are able to provide particle identification (PID) via energy-loss measurements.

The data analysis is carried out using a sample of $\sim$ 250 million minimum-bias pp collisions at $\sqrt{s}$ $=$ 7 TeV collected during 2010.

During the data-taking period, the luminosity at the interaction point was kept in the range $0.6{-}1.2\times 10^\mathrm{29}$ cm$^\mathrm{{-2}}$ s$^\mathrm{{-1}}$. Runs with a mean pile-up probability per event larger than 2.9 % are excluded from the analysis. The vertex of each collision is required to be within $\pm$10 cm of the detector’s centre along the beam direction. The event vertex range is selected to optimize the reconstruction efficiency of particle tracks within the ITS and TPC acceptance.

### Particle selections

The resonances are reconstructed via their hadronic decay channel, shown in Table 1 together with the branching ratio (BR).

For ${\Sigma }$(1385), all four charged species (${\Sigma }$(1385)$^{+}$, ${\Sigma }$(1385)$^{-}$, ${\overline{\Sigma }}$(1385)$^{-}$ and ${\overline{\Sigma }}$(1385)$^{+}$) are measured separately.

$${\Xi (1530)^{0}}$$ is measured together with its antiparticle ($${\overline{\Xi } (1530)^{0}}$$) due to limited statistics. Therefore in this paper, unless otherwise specified, $${\Xi (1530)^{0}}$$ $$\equiv ($$ $${\Xi (1530)^{0}}$$+$${\overline{\Xi } (1530)^{0}}$$ $$)/2$$.

Note that, for brevity, antiparticles are not listed and the selection criteria, described in the following, are discussed for particles; equivalent criteria hold for antiparticles.

Several quality criteria, summarized in Table 2, are used for track selection.

Charged pions from the strong decay of both ${\Sigma }$(1385) and ${\Xi (1530)^{0}}$ are not distinguishable from primary particles and therefore primary track selections are used. They are requested to have a distance of closest approach (DCA) to the primary interaction vertex of less than 2 cm along the beam direction and a DCA in the transverse plane smaller than 7 $\sigma _{\mathrm {DCA}}$($p_{\mathrm{T}}$), where $\sigma _{\mathrm {DCA}}$($p_{\mathrm{T}}$) $=$ (0.0026 $+$ 0.0050 GeV$/c$ $\times$ $p_{\mathrm{T}}$ $^{-1}$) cm is the parametrization which accounts for the $p_{\mathrm{T}}$-dependent resolution of the DCA in the transverse plane [18]. Primary tracks are also required to have at least one hit in one of the two innermost layers of the ITS (silicon pixel detector, SPD) and at least 70 reconstructed clusters in the TPC out of the maximum 159 available, which keeps the contamination from secondary and fake tracks small, while ensuring a high efficiency and good d$E$/d$x$ resolution.

Tracks close to the TPC edge or with transverse momentum $p_{\mathrm{T}}$ $< 0.15$ GeV$/c$ are rejected because the resolution of track reconstruction deteriorates.

In the ${\Sigma }$(1385) analysis, PID is implemented for ${\uppi ^\pm }$ and p from ${\Lambda }$.

Particles are identified based on a comparison of the energy deposited in the TPC drift gas and an expected value computed using a Bethe–Bloch parametrization [19]. The filter is set to 3 $\sigma _\mathrm{TPC}$, where $\sigma$ is the resolution estimated by averaging over reconstructed tracks. An averaged value of $\sigma _\mathrm{TPC}$ $=$ 6.5 % is found over all reconstructed tracks [20].

PID selection criteria are not applied in the ${\Xi }$(1530) analysis as the combinatorial background is sufficiently removed through topological selection.

$\Lambda$ produced in the decay of ${\Sigma }$(1385) decays weakly into $\pi ^{-}$p with ${c}\tau =$ 7.89 cm [17]. These pions and protons do not originate from the primary collision vertex, and thus they are selected using a DCA to the interaction point greater than 0.05 cm. At least 70 reconstructed clusters in the TPC are requested for these tracks. Further selection criteria to identify ${\Lambda }$ are applied on the basis of the decay topology as described in [19]. Selection criteria for ${\Lambda }$ used in the ${\Sigma }$(1385) analysis are summarized in Table 3.

$\Xi ^{-}$ produced in the decay of the ${\Xi (1530)^{0}}$ decays weakly into $\Lambda \pi ^{-}$ with ${c}\tau =$ 4.91 cm [17]. Pions are selected from tracks with a DCA to the interaction point greater than 0.05 cm. Pions and protons from $\Lambda$ are required to have a DCA to the interaction point greater than 0.04 cm. All pions and protons are requested to have at least 70 reconstructed clusters in the TPC. Decay topologies for $\Xi ^{-}$ and $\Lambda$ are used as described in [19]. Selection criteria are summarized in Table 4.

**Table 3:** Selection criteria used in the ${\Sigma }$(1385) analysis. *PV* primary vertex, $R_{r}$ transverse radius of the decay vertex

$|y_\mathrm{{\Sigma }^{*}}|$	$<$0.5
DCA of ${\Lambda }$ decay products to PV	>0.05 cm
DCA between ${\Lambda }$ decay products	$<$1.6 standard deviations
DCA of ${\Lambda }$ to PV	$<$0.3 cm
${\Lambda }$ cosine of pointing angle	>0.99
${\Lambda }$ fiducial volume (R$_{r}$)	$1.4<$ R$_{r}<$ 100 cm
${\Lambda }$ invariant mass window	$m_\mathrm{{PDG}}$ $\pm$ 10 MeV/$c^{\mathrm 2}$

All these criteria are optimized to obtain maximum signal significance. Values for the significance are presented in Sect. 2.2.3.

### Signal extraction

#### Combinatorial background and event-mixing

Due to their very short lifetime of a few fm/$c$, resonance decay products originate from a position that is indistinguishable from the primary vertex. Thus, the computation of invariant mass distributions for potential resonance decay candidates has significant combinatorial background that has to be subtracted to ensure reliable yield determination.

**Fig. 1:**
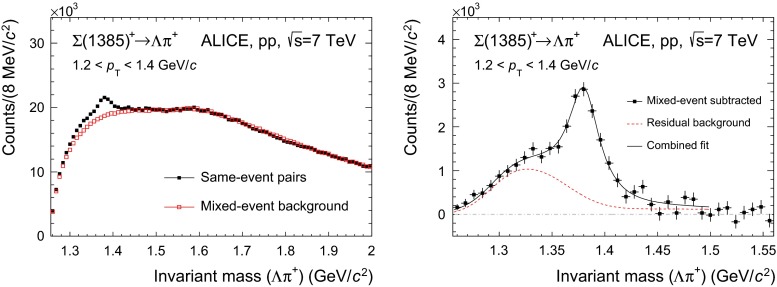
*Left panel* The ${\Lambda }$ ${\uppi ^+}$ invariant mass distribution in $\left| y \right|$ $<$ 0.5 for the transverse momentum bin 1.2 $<$ $p_{\mathrm{T}}$ $<$1.4 GeV$/c$  in pp collisions at $\sqrt{s}$ $=$ 7 TeV. The background shape estimated using pairs from different events (event-mixing) is shown as *open red squares*. The mixed-event background is normalized in the range 1.56 $<M<$ 2.0 GeV$/c^2$, where $M$ is the ${\Lambda }$ ${\uppi ^+}$ invariant mass. *Right panel* The invariant mass distribution after mixed-event background subtraction for 1.2 $<$ $p_{\mathrm{T}}$ $<$1.4 GeV$/c$. The *solid curve* is the result of the combined fit (see text for details) and the *dashed lines* describes the residual background

**Fig. 2:**
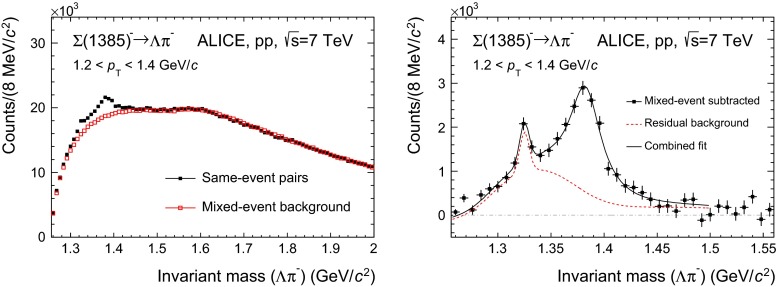
Same as Fig. 1 but for ${\Sigma }$(1385)$^{-}$ $\longrightarrow$ ${\Lambda }$ $+$ ${\uppi ^-}$. Note the peak at around the $\Xi (1321)^{-}$ mass, which is absent in Fig. 1

**Fig. 3:**
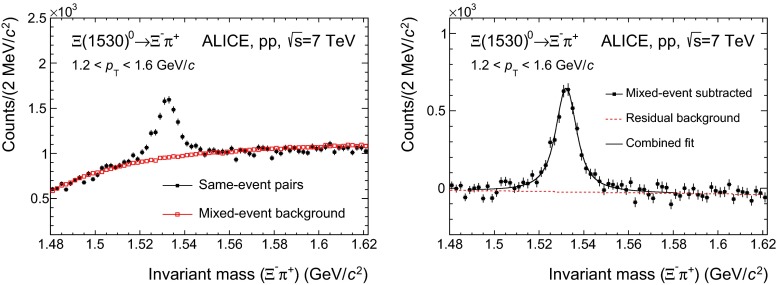
*Left panel* The $${\Xi }$$ $$^{-}$$ $${\uppi ^+}$$ invariant mass distribution in $$\left| y \right| $$ $$<$$ 0.5 for the transverse momentum bin $$1.2<$$ $$p_{\mathrm{T}}$$ $$~<1.6$$ GeV$$/c$$  in pp collisions at $$\sqrt{s}$$ $$=$$ 7 TeV. The background shape estimated using pairs from different events (event-mixing) is shown as *open red squares*. The mixed-event background is normalized in the range 1.49 $$<M<$$ 1.51 GeV$$/c^2$$. *Right panel* The invariant mass distribution after mixed-event background subtraction for $$1.2<$$ $$p_{\mathrm{T}}$$ $$ <1.6$$ GeV$$/c$$. The *solid curve* is the result of the combined fit and the *dashed line* describes the residual background

This is shown in the left panels of Figs. 1 and 2 (for ${\Sigma }$(1385)$^{+}$ and ${\Sigma }$(1385)$^{-}$, respectively) and Fig. 3 (for the ${\Xi (1530)^{0}}$).

Figures similar to Figs. 1 and 2 are obtained for the antiparticles ${\overline{\Sigma }}$(1385)$^{-}$ and ${\overline{\Sigma }}$(1385)$^{+}$. In Fig. 2 the peak from $\Xi ^{-}\longrightarrow \Lambda + \pi ^{-}$ is visible.

**Table 4:** Same as Table 3 but for the ${\Xi }$(1530) analysis

$|y_\mathrm{{\Xi }^{*}}|$	$<$0.5
DCA of ${\Lambda }$ decay products to PV	>0.04 cm
DCA between ${\Lambda }$ decay products	$<$1.6 standard deviations
DCA of ${\Lambda }$ to PV	>0.07 cm
${\Lambda }$ cosine of pointing angle	>0.97
${\Lambda }$ fiducial volume (R$_{r}$)	$0.8<$ R$_{r}<$ 100 cm
$\Lambda$ invariant mass window	$m_\mathrm{{PDG}}$ $\pm$ 6 MeV/$c^{\mathrm 2}$
DCA of pion (from $\Xi ^{-}$) to PV	>0.05 cm
DCA between $\Xi ^{-}$ decay products	$<$1.6 standard deviations
$\Xi ^{-}$ cosine of pointing angle	>0.97
$\Xi ^{-}$ fiducial volume (R$_{r}$)	$0.8<$ R$_{r}<$ 100 cm
$\Xi ^{-}$ invariant mass window	$m_\mathrm{{PDG}}$ $\pm$ 6 MeV/$c^{\mathrm 2}$

The combinatorial background distributions are obtained and subtracted from the invariant mass distribution by means of a mixed-event technique, in which a reference background distribution is built with uncorrelated candidates from different events. To avoid mismatch due to different acceptances and to ensure a similar event structure, only tracks from events with similar vertex positions $z$ ($\Delta z <1$ cm) and track multiplicities $n$ ($\Delta n <10$) are mixed. In order to reduce statistical uncertainties, each event is mixed with several other events (5 in the ${\Sigma }$(1385) analysis and $>20$ in the ${\Xi (1530)^{0}}$ analysis), so that the total number of entries in the mixed-event invariant mass distribution is higher than the total number of entries in the distribution from the same event. Thus the mixed-event distribution needs to be scaled before it can be used to describe the background in the same-event distribution. For ${\Sigma }$(1385), the regions for the normalization of the mixed-event distribution are selected in the rightmost part of the invariant mass window, where the residual background is absent (see Sect. 2.2.2 for a description of the residual background). These regions are different for the different $p_{\mathrm{T}}$ bins, ranging from 1.48 $<M<$ 2.0 GeV$/c^2$, for the lowest $p_{\mathrm{T}}$ bin, to 1.95 $<M<$ 2.0 GeV$/c^2$, for the highest $p_{\mathrm{T}}$ bin ($M$ being the invariant mass of ${\Sigma }$(1385) and 2.0 GeV$/c^2$ being the upper extreme of the invariant mass window). The reason for this $p_{\mathrm{T}}$-dependent choice is due to the reach of the residual background, which is higher in invariant mass for higher $p_{\mathrm{T}}$. Fixed regions, 1.6 $<M<$ 1.8 GeV$/c^2$ and 1.8 $<M<$ 2.0 GeV$/c^2$, have also been tried, giving a systematic uncertainty of $\sim$ 1 %. For ${\Xi (1530)^{0}}$ a fixed region 1.49 $<M<$ 1.51 GeV$/c^2$, just at the left of the signal, is selected. A fixed region can be selected because for all $p_{\mathrm{T}}$ intervals the background shape is similar and the invariant mass resolution on the reconstructed peak is the same. The uncertainty in the normalization ($\sim$ 1 %), which is included in the quoted systematic uncertainty for signal extraction, is estimated by using another normalization region, 1.56 $<M<$ 1.58 GeV$/c^2$, just at the right of the signal. The open squares in the left panels of Figs. 1, 2 and 3 correspond to the properly scaled mixed-event invariant mass distribution.

The right panels show the signals for each resonance after the mixed-event combinatorial background is subtracted.

#### Residual correlated background

The mixed-event technique removes only uncorrelated background pairs in the invariant mass spectrum. The consequence is that residual correlations near the signal mass range are not subtracted by the mixed-event spectrum and correlated background pairs remain [21]. This is especially dominant for ${\Sigma }$(1385) (see Figs. 1, 2, right), for which the correlated residual background takes contributions from two dominant sources:

- Type A: correlated $\Lambda \pi$ pairs coming from the decays of other particles which have ${\Lambda }$ and $\pi$ among the decay products.
- Type B: correlated $\Lambda \pi$ pairs which come from the dynamics of the collision and are not removed from the subtraction of the mixed-event background.

All these contributions are present in the MC, albeit with potentially incorrect proportions. Thus, simulations are used to determine the shapes of such contributions in invariant mass space and then these contributions are renormalized using data, as described later.

All the sources of contamination of Type A, which can potentially produce correlated $\Lambda \pi$ pairs, are listed in Table 5. A similar scheme, not discussed for sake of brevity, is valid for the antiparticles (e.g. the $\overline{\Xi }^{+}$ $\longrightarrow$ ${\overline{\Lambda }}$ ${\uppi ^+}$ decay channel affects the reconstruction of ${\overline{\Sigma }}$(1385)$^{+}$). Only sources A1, A5 and A6 in Table 5 give a significant contribution to the correlated residual background of Type A. This is discussed in the following.

**Table 5:** Potential sources of contamination in the reconstruction of ${\Sigma }$(1385). Checkmarks show which species is potentially affected. Checkboxes further indicate whether the source gives a significant contamination (see text). A similar scheme, not shown for sake of brevity, is valid for the antiparticles

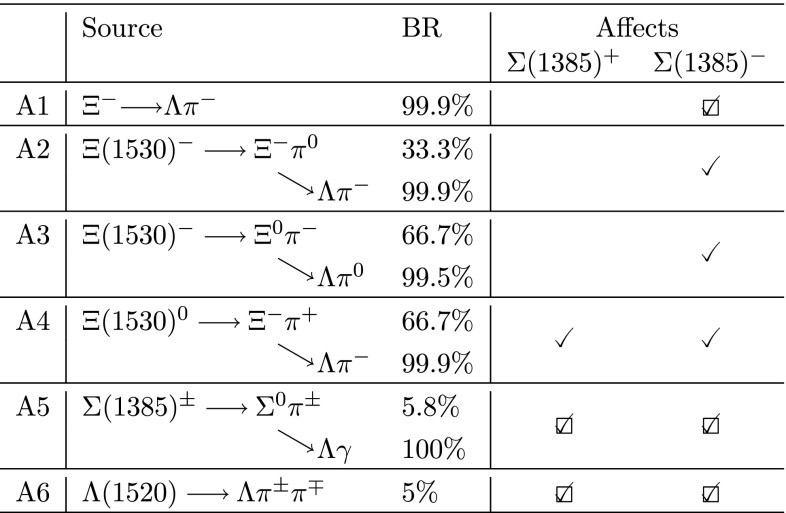	

Source A1 in Table 5 is due to the primary $\Xi ^{-}$ which decays weakly to $\Lambda \pi ^{-}$, affecting the reconstruction of ${\Sigma }$(1385)$^{-}$. Since the $\Xi ^{-}$ hyperon is metastable, it shows up in the $\Lambda \pi ^{-}$ invariant mass spectrum as a very narrow peak at around the $\Xi ^{-}$ mass, $M_\mathrm{{\Xi ^{-}}}=$ 1321.71 MeV/$c^{\mathrm 2}$ [17], just on the left tail of the ${\Sigma }$(1385)$^{-}$ signal. The $\Xi ^{-}$ peak is clearly seen in Fig. 2. This contribution, which is expected to be important since the yield of $\Xi ^{-}$ is comparable to the yield of ${\Sigma }$(1385)$^{-}$, is in fact suppressed, by an order of magnitude, because the filter on the DCA to the primary vertex of both $\Lambda$ and $\pi$ filters out most of the ${\Lambda }$ $\pi$ pairs from $\Xi ^{-}$. Indeed, the filter on the DCA to the primary vertex is optimized for the ${\Sigma }$(1385) decay products, which are not distinguishable from primary particles (see Sect. 2.1), whereas $\Lambda$ and $\pi$ from $\Xi ^{-}$ come from a secondary vertex, centimetres away from the primary vertex. Only a small percentage of the $\Xi ^{-}$ yield survives the filter on the DCA. Source A1 is taken into account by adding a Gaussian function, with the mean value fixed to the $\Xi ^{-}$ mass and the width and normalization left free, to the combined fit of the invariant mass spectrum in the reconstruction of ${\Sigma }$(1385)$^{-}$. The contamination from $\Xi ^{-}$ reaches about 5–10 % of the raw ${\Sigma }$(1385)$^{-}$ signal and varies little with $p_{\mathrm{T}}$.

Sources A2, A3 and A4 give a negligible contribution. Sources A2 and A3 are due to the hadronic decay channels of $\Xi (1530)^{-}$, with BR $=$ 33.3 % and BR $=$ 66.7 %, respectively^1^, and, like A1, affect only the ${\Sigma }$(1385)$^{-}$ reconstruction. Source A4 is due to $\Xi (1530)^{0}$ and potentially affects the reconstruction of both ${\Sigma }$(1385)$^{+}$ and ${\Sigma }$(1385)$^{-}$, since it involves two opposite-sign pions. The same topological considerations hold for A2 as they do for A1, since it involves a $\Xi ^{-}$. Indeed, this $\Xi ^{-}$ comes from the strong decay of $\Xi (1530)^{-}$, therefore it is practically not distinguishable from the (primary) $\Xi ^{-}$ in A1. Unlike contribution A1, a further suppression, of about an order of magnitude with respect to A1, comes from both the smaller yield of $\Xi (1530)^{-}$ with respect to the primary $\Xi ^{-}$, and the BR of the $\Xi (1530)^{-}\rightarrow \Xi ^{-}\pi ^{0}$ channel. This further suppression makes contribution A2 practically negligible. Similar conclusions hold for contributions A3 and A4.

Source A5 in Table 5 is related to the second $\Sigma (1385)$ decay channel, $\Sigma (1385)^{\pm }\rightarrow \Sigma ^{0}\pi ^{\pm }$ (BR $=$ $5.8$ %^2^), with $\Sigma ^{0}\rightarrow \Lambda \gamma$ (BR $\simeq$ 100 % [17]). $\Lambda$ from $\Sigma ^{0}$ is paired with $\pi ^{\pm }$ from $\Sigma (1385)^{\pm }$. This gives a Gaussian-like peak at around 1.306 GeV$/c^2$, with a width of $\sim$ 0.059 GeV$/c^2$ (FWHM). This peak is used in the combined fit to the signal (see below) with a relative normalization with respect to the signal which accounts for the ratio ($=$0.067) between the BR ($=$5.8 %) for the $\Sigma (1385)^{\pm }\rightarrow \Sigma ^{0}\pi ^{\pm }$ channel and the BR ($=$87 %) for the $\Sigma (1385)^{\pm }\rightarrow \Lambda \pi ^{\pm }$ channel.

Source A6 in Table 5 is due to the $\Lambda (1520)\rightarrow \Lambda \pi ^{\pm }\pi ^{\mp }$ channel (BR $=$ $5$ %^3^). The positive (negative) pion, paired with $\Lambda$, produces a Gaussian-like peak, which contaminates the invariant mass distribution of ${\Sigma }$(1385)$^{+}$ (${\Sigma }$(1385)$^{-}$). This peak is centred at $\sim$ 1.315 GeV$/c^2$ and has a width of $\sim$ 0.076 GeV$/c^2$ (FWHM). The peak is used in the combined fit to the signal. The normalization of the peak is kept free in the fit since the $\Lambda$(1520) yield is not measured. The contamination from $\Lambda$(1520) decreases with increasing $p_{\mathrm{T}}$, ranging from about 75 % of the raw ${\Sigma }$(1385)$^{-}$ signal in the first $p_{\mathrm{T}}$ interval, down to 0 for $p_{\mathrm{T}}$ $>$ 4 GeV$/c$.

A third-degree polynomial is used to fit the residual background of Type B in the MC. The fit to MC data is performed in the region from 1.26 GeV$/c^2$ (just left of the signal region) to the lower edge of the event-mixing normalization region. The fitting function is then normalized to the residual background in real data; the normalization is done in the region from 1.46 GeV$/c^2$ (just right of the signal region) to the lower edge of the event-mixing normalization region, where other sources of contamination are absent. The lower point of the normalization region is the same for all $p_{\mathrm{T}}$ intervals since the mean, the width and the invariant mass resolution on the reconstructed peak stay the same over all the $p_{\mathrm{T}}$ range considered. Comparable results are obtained from using different event generators (PYTHIA 6.4, tune Perugia 0 [22], and PHOJET [23]) and other degrees for the polynomial (second and fourth). The differences of about 2 % are included in the systematic uncertainties.

The invariant mass distribution is fitted with a combined fit function: a (non-relativistic) Breit–Wigner peak plus the functions that make up the residual background (Figs. 1, 2, right). The Breit–Wigner width $\Gamma$ is kept fixed to the PDG value to improve the stability of the fit.

For ${\Xi (1530)^{0}}$, the residual background after the mixed-event background subtraction is fitted with a first-degree polynomial. The fitting procedure is done in three stages. First, the background is fitted alone from 1.48 to 1.59 GeV$/c^2$ while excluding the $\Xi$(1530)$^0$ mass region from 1.51 to 1.56 GeV$/c^2$. Second, a combined fit for signal and background is performed over the full range with the background polynomial fixed to the results from the first fit stage; a Voigtian function—a convolution of Breit–Wigner and Gaussian functions—is used for the signal. The Gaussian part accounts for detector resolution. Third, a fit is redone over the full range again with all parameters free but set initially to the values from the second stage.

#### Counting signal and signal characteristics

The above procedure is applied for 10 (8) $p_{\mathrm{T}}$ bins for ${\Sigma }$(1385) (${\Xi (1530)^{0}}$), from 0.7 to 6.0 (0.8 to 5.6) GeV$/c$. For ${\Sigma }$(1385), the fit is repeated leaving the Breit–Wigner width $\Gamma$ free to move, and, for each $p_{\mathrm{T}}$ interval, the difference in the yield is included in the systematic uncertainties ($\sim$ 4 % maximum contribution). The widths of both ${\Sigma }$(1385) and ${\Xi (1530)^{0}}$ are consistent with the PDG values for all $p_{\mathrm{T}}$ intervals. In the ${\Sigma }$(1385)$^{-}$ analysis, a Gaussian function, centred at 1.321 GeV$/c^2$ and with a starting value for the width of 2 MeV/$c^{\mathrm 2}$, is used to help the combined fit around the $\Xi$(1321)$^{-}$ peak (Fig. 2). The value of 2 MeV/$c^{\mathrm 2}$ is obtained from the analysis of $\Xi$(1321)$^{-}$ [19] and is related to the mass resolution. Since the ${\Sigma }$(1385) mass binning of 8 MeV/$c^{\mathrm 2}$, which is optimised for the $\chi ^{2}$ of the combined fit, is larger than the mass resolution, only a rough description of the $\Xi$(1321)$^{-}$ peak is possible. For ${\Xi (1530)^{0}}$, the standard deviation of the Gaussian component of the Voigtian peak is found to be $\sim$ 2 MeV/$c^{\mathrm 2}$, which is consistent with the detector resolution, as obtained from the MC simulation. At low $p_{\mathrm{T}}$, the fitted mass values for ${\Sigma }$(1385) are found to be slightly lower (by $\sim$ 5 MeV/$c^{\mathrm 2}$) than the PDG value, which is attributed to imperfections in the corrections for energy loss in the detector material. For ${\Xi (1530)^{0}}$, the reconstructed masses are found to be in agreement with the PDG value within the statistical uncertainties.

The raw yields $N^\mathrm{RAW}$ are obtained by integrating the Breit–Wigner function. As an alternative, $N^\mathrm{RAW}$ is calculated by integrating the invariant mass histogram after the subtraction of the event-mixing background and subtracting the integral of the residual background (bin-counting method). The difference between the two methods of integration is lower than 2 % on average.

Significance values (defined as $S/\sqrt{S+B}$, where $S$ is the signal and $B$ the background) for ${\Sigma }$(1385)$^+$ (${\Xi (1530)^{0}}$) are found to be 16.6 (16.5) in the lowest $p_{\mathrm{T}}$ interval, and 20.9 (22.8) in the highest $p_{\mathrm{T}}$ interval, and reached 24.2 (52.4) in the intermediate $p_{\mathrm{T}}$ interval. Significance values comparable to those of ${\Sigma }$(1385)$^+$ are obtained for the other ${\Sigma }$(1385) species.

### Correction and normalization

In order to extract the baryon yields, $N^\mathrm{RAW}$ are corrected for BR, the geometrical acceptance ($A$), the detector efficiency ($\epsilon$) and the correction factor which accounts for the GEANT3 overestimation of the $\bar{\mathrm{p}}$ cross sections ($\epsilon _\mathrm{GEANT3/FLUKA}$) [24]

1 $$\begin{aligned} N^\mathrm{cor}(p_{\mathrm{T}})=\frac{N^\mathrm{RAW}(p_{\mathrm{T}})}{\mathrm{BR}\; (A\times \epsilon )(p_{\mathrm{T}})}\; \epsilon _\mathrm{GEANT3/FLUKA}(p_{\mathrm{T}}). \end{aligned}$$

**Fig. 4:**
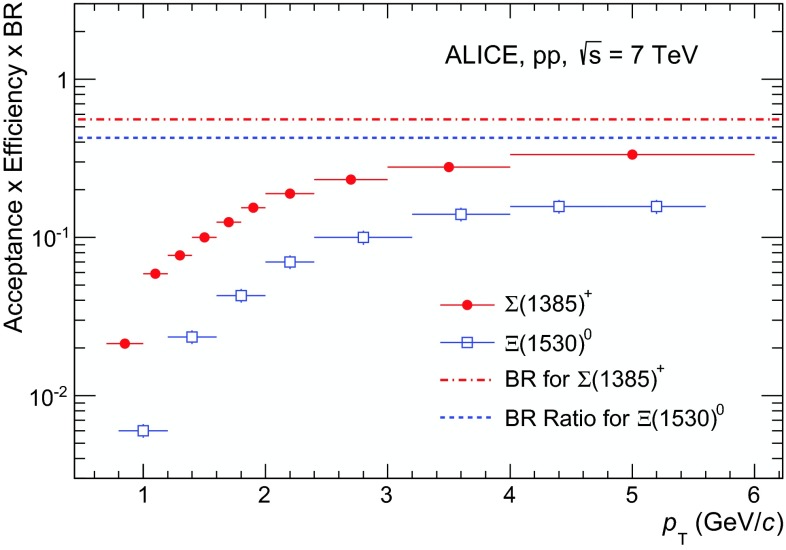
The product of acceptance, efficiency and branching ratio of ${\Sigma }$(1385)$^+$ and ${\Xi }$(1530)$^0$, obtained with PYTHIA 6.4 [22] and GEANT3 [25], as function of $p_{\mathrm{T}}$ in $\left| y \right|$ $<$0.5. Only statistical uncertainties are reported. The *dashed*- and the *dash-dotted lines* indicate the overall branching ratio for the two reconstruction channels

The product of acceptance and efficiency ($A \times \epsilon$) is determined from MC simulations with the PYTHIA 6.4 event generator (tune Perugia 0 [22]) and a GEANT3-based simulation of the ALICE detector response [25]. The $\epsilon _\mathrm{GEANT3/FLUKA}$ correction factor is equal to 0.99 for the protons from ${\Sigma }$(1385)$^{\pm }$ and ${\Xi (1530)^{0}}$ and ranges from 0.90 to 0.98, from the lowest to the highest $p_{\mathrm{T}}$ interval, for the antiprotons from ${\overline{\Sigma }}$(1385)$^{\pm }$ and ${\overline{\Xi } (1530)^{0}}$. About $200\times 10^6$ Monte-Carlo events, with the same vertex distribution as for the real events, were analysed in exactly the same way as for the data. The $A\times \epsilon$ is determined from MC simulations as the ratio of the number of reconstructed resonances to the number of those generated in $\left| y \right|$ $<0.5$, differentially as a function of transverse momentum, as shown in Fig. 4.

The drop in efficiency at low $p_{\mathrm{T}}$ is due to the loss of slow pions involved in the decay chain. As a cross-check, the efficiency $\times$ acceptance has also been assessed with PHOJET [23] as event generator. The relative difference of the resulting $A \times \epsilon$, averaged over the various $p_{\mathrm{T}}$ intervals, is below 1 %.

Finally, corrections for the trigger inefficiency ($\epsilon _\mathrm{trigger}$) and the loss of candidates outside of the $z$-vertex range ($\epsilon _\mathrm{vert}$) are applied via

2 $$\begin{aligned} \frac{1}{N_\mathrm{INEL}}\frac{\mathrm {d}^2N}{\mathrm {d}y\mathrm {d}p_{\mathrm{T}}} = \frac{N^\mathrm{cor}(p_{\mathrm{T}})}{\Delta y \Delta p_{\mathrm{T}}} \; \frac{\epsilon _\mathrm{trigger} }{\epsilon _\mathrm{vert} }\; \frac{1}{N_ \mathrm{MB}}, \end{aligned}$$

where $N^{\mathrm {cor}}$ and $N_ {\mathrm {MB}}$ are the number of reconstructed ${\Sigma }$(1385) or ${\Xi }$(1530) and the total number of minimum bias triggers, respectively. $\Delta$ $y$ and $\Delta$ $p_{\mathrm{T}}$ are the rapidity window width and the $p_{\mathrm{T}}$ bin width, respectively. The trigger selection efficiency for inelastic collisions $\epsilon _{\mathrm {trigger}}$ is equal to $0.852 ^{+0.062} _{-0.030}$ [26]. The loss of resonances due to the trigger selection, estimated by MC simulations, is negligible, less than 0.2 %. The $\epsilon _\mathrm{vert}$ correction factor accounts for resonance losses ($\sim$ 7 %) due to the requirement to have a primary vertex $z$ position in the range $\pm$10 cm.

### Systematic uncertainties of $p_{\mathrm{T}}$ spectra

Two types of systematic uncertainties in the particle spectra are considered: $p_{\mathrm{T}}$-dependent systematic uncertainties, which are due to the selection efficiency and signal extraction at a given $p_{\mathrm{T}}$, and $p_{\mathrm{T}}$-independent uncertainties due to the normalization to inelastic collisions and other corrections.

The minimum and maximum values of the major contributions to the point-to-point systematic uncertainties are listed in Table 6.

**Table 6:** Summary of the systematic uncertainties in the ${\Sigma }$(1385) and $\Xi (1530)$ differential yield, $\mathrm{d}^2N/(\mathrm{d}y \mathrm{d}p_{\mathrm{T}})$

Source of uncertainty	${\Sigma }$(1385)	$\Xi$(1530)
Point-to-point		
Signal extraction	8–11	5–6
Tracks selection	7	1–3
Topological selection	6–7	3–4
PID efficiency	4–6	–
$p_{\mathrm{T}}$-independent		
INEL normalization	$^{+7.3}_{-3.5}$	$^{+7.3}_{-3.5}$
Material budget	4	4
GEANT3/FLUKA correction	2	2
Branching ratio	1.5	–

The uncertainties introduced by tracking, topology selection and PID are obtained by varying the selection criteria for the decay products. To this purpose, the selection criteria listed in Tables 2, 3 and 4 are changed by a certain amount which varies the raw yield in real data by $\pm$10 %. The maximum difference between the default yield and the alternate value obtained by varying the selection, is taken as systematic uncertainty. The uncertainties introduced by the signal extraction come from several sources: normalization of the event-mixing background, fitting function and range of the residual background, signal fitting and integration. For ${\Sigma }$(1385), the contamination from the $\Lambda$(1520) introduced the largest contribution ($\sim$ 8 %). All the sources are combined by summing in quadrature the uncertainties for each $p_{\mathrm{T}}$.

Among the $p_{\mathrm{T}}$-independent uncertainties, the INEL normalization leads to a +7.3 % and $-$3.5 % uncertainty [26], the determination of the material thickness traversed by the particles (material budget) introduces a 4 % uncertainty and the use of FLUKA [27, 28] to correct the antiproton absorption cross section in GEANT3 leads to a further 2 % uncertainty [24]. For ${\Sigma }$(1385), a further 1.5 % comes from the uncertainty in the branching ratio. A summary of the $p_{\mathrm{T}}$-independent uncertainties is presented in Table 6.

## Results

**Fig. 5:**
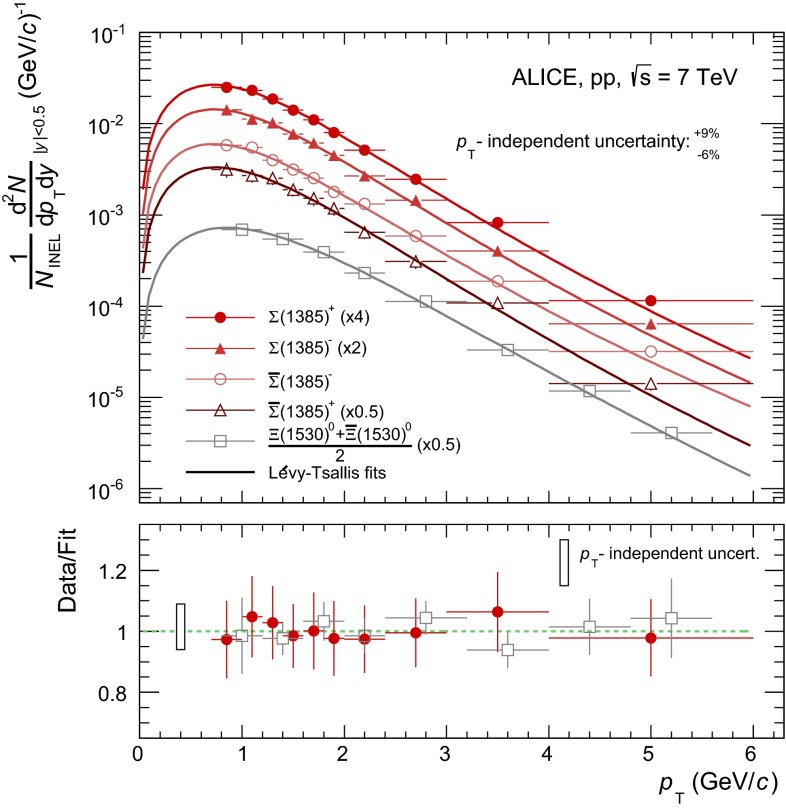
Inelastic baryon yields, $\mathrm{d}^2N/(\mathrm{d}y \mathrm{d}p_{\mathrm{T}})$, per $p_{\mathrm{T}}$ interval per unit rapidity for ${\Sigma }$(1385) and ${\Xi (1530)^{0}}$. Statistical and systematic uncertainties are summed in quadrature, excluding the $p_{\mathrm{T}}$-independent uncertainties, which affect only the overall normalization of the spectra and are not considered in the fit. Spectra are fitted with a Lévy–Tsallis function. The ratio data/fit is shown in the *lower panel*. For the sake of visibility, only ${\Sigma }$(1385)$^{+}$ is shown in the *lower panel*, but similar ratios have been obtained for the other three ${\Sigma }$(1385) species. For the ratio, the integral of the fitting function in each corresponding $p_{\mathrm{T}}$ interval is considered. Spectra points are represented at the centre of the $p_{\mathrm{T}}$ interval

The corrected baryon yields per $p_{\mathrm{T}}$ interval per unit rapidity (1/$N_\mathrm{INEL}$ $\times$ $\mathrm{d}^2N/(\mathrm{d}y \mathrm{d}p_{\mathrm{T}})$) are shown in Fig. 5. They cover the ranges 0.7 $<$ $p_{\mathrm{T}}$ $<$ 6.0 GeV$/c$ for ${\Sigma }$(1385) and 0.8 $<$ $p_{\mathrm{T}}$ $<$ 5.6 GeV$/c$  for ${\Xi (1530)^{0}}$.

**Table 7:** Parameters extracted from the Lévy–Tsallis (LT) fits (Eq. 3) to the transverse momentum spectra. The values of d$N$/d$y$ are calculated using the spectra in the measured range and the extrapolation of the fitted Lévy–Tsallis function outside the measured range. Systematic uncertainties quoted here are the ones derived from Lévy–Tsallis fit only (see text)

Baryon	d$N$/d$y$ (LT) ($\times$10$^{-3}$)	$C$ (MeV)	$n$	$\langle p_{\mathrm{T}}\rangle$ (LT) (GeV$/c$)	$\chi ^{2}/n\mathrm {d}f$
${\Sigma }$(1385)$^{+}$	9.8 $\pm$ 0.2 $\pm$ 0.9	301 $\pm$ 39 $\pm$ 15	9.0 $\pm$ 2.9 $\pm$ 0.5	1.17 $\pm$ 0.02 $\pm$ 0.03	1.13/7
${\Sigma }$(1385)$^{-}$	10.6 $\pm$ 0.2 $\pm$ 1.1	308 $\pm$ 39 $\pm$ 20	9.1 $\pm$ 3.2 $\pm$ 0.8	1.17 $\pm$ 0.02 $\pm$ 0.03	1.71/7
${\overline{\Sigma }}$(1385)$^{-}$	9.0 $\pm$ 0.2 $\pm$ 0.9	307 $\pm$ 40 $\pm$ 15	9.8 $\pm$ 3.7 $\pm$ 0.8	1.18 $\pm$ 0.02 $\pm$ 0.04	1.19/7
${\overline{\Sigma }}$(1385)$^{+}$	10.0 $\pm$ 0.2 $\pm$ 1.1	294 $\pm$ 43 $\pm$ 17	8.9 $\pm$ 3.5 $\pm$ 0.6	1.18 $\pm$ 0.02 $\pm$ 0.04	1.53/7
${\Xi (1530)^{0}}$	2.48 $\pm$ 0.07 $\pm$ 0.24	404 $\pm$ 20 $\pm$ 21	16.9 $\pm$ 3.9 $\pm$ 1.9	1.33 $\pm$ 0.02 $\pm$ 0.05	2.24/5

The vertical error bars in Fig. 5 represent the sum in quadrature of the statistical and systematic uncertainties, excluding the $p_{\mathrm{T}}$-independent uncertainties, which affect only the normalization.

All spectra are fitted with a Lévy–Tsallis function [29], which is used for most of the identified particle spectra in pp collisions [19, 20, 30–32],

3 $$\begin{aligned}&\frac{1}{N_\mathrm{INEL}}\frac{\mathrm {d}^{2}N}{\mathrm {d}y\mathrm {d}p_{\mathrm{T}}} \nonumber \\&\quad = \frac{(n-1)(n-2)}{nC [nC + m_{0}(n-2)]} \frac{\mathrm {d}N}{\mathrm {d}y} \; {p_{\mathrm{T}}} \left( 1+ \frac{m_{\mathrm {T}}-m_{0}}{nC}\right) ^{-n} , \end{aligned}$$

where $m_\mathrm{T}=\sqrt{m_{0}^2+p_{\mathrm{T}}^2}$ and $m_{0}$ denotes the PDG particle mass. This function, quantified by the inverse slope parameter $C$ and the exponent parameter $n$, describes both the exponential shape of the spectrum at low $p_{\mathrm{T}}$ and the power law distribution at large $p_{\mathrm{T}}$. The parameter d$N$/d$y$ represents the particle yield per unit rapidity per INEL event. d$N$/d$y$, $C$ and $n$ are the free parameters considered for this function. Table 7 presents the parameter outcome of the Lévy–Tsallis fit, together with the mean transverse momentum, $\langle p_{\mathrm{T}}\rangle$, and the reduced $\chi ^{2}$.

The values of d$N$/d$y$ in Table 7 are obtained by adding the integral of the experimental spectrum in the measured range and the extrapolations with the fitted Lévy–Tsallis function to both $p_{\mathrm{T}}$ $=0$ and high $p_{\mathrm{T}}$. The contribution of the low-$p_{\mathrm{T}}$ extrapolation to the total d$N$/d$y$ is $\sim$ 30 % for both ${\Sigma }$(1385) and ${\Xi (1530)^{0}}$. The contribution of the high-$p_{\mathrm{T}}$ extrapolation is negligible.

For each species considered here, such a composite d$N$/d$y$ differs very little ($<$ 1 %) from the value of d$N$/d$y$ as the first free parameter returned by the fit, i.e. from the integration of the fit function from 0 to infinity.

In order to obtain the systematic uncertainty on the parameters of the Lévy–Tsallis fit (d$N$/d$y$, $C$ and $n$) and on the mean transverse momentum ($\langle p_{\mathrm{T}}\rangle$), the Lévy–Tsallis fit is repeated for each $p_{\mathrm{T}}$ spectrum obtained by varying separately the selection criteria in each source of systematic uncertainties. Only statistical uncertainties on the points of the $p_{\mathrm{T}}$ spectrum are used for the fit. The values for d$N$/d$y$, $C$, $n$ and $\langle p_{\mathrm{T}}\rangle$, obtained for each source, are compared to those from the fit to the reference $p_{\mathrm{T}}$ spectrum, obtained with default selection criteria. The fit to the reference $p_{\mathrm{T}}$ spectrum is also done with statistical uncertainties only. The statistically significant differences are summed in quadrature to contribute to the overall systematic uncertainties on d$N$/d$y$, $C$, $n$ and $\langle p_{\mathrm{T}}\rangle$.

Although the Lévy–Tsallis function describes the spectra both at low and at large $p_{\mathrm{T}}$, other functions (e.g. $m_{\mathrm {T}}$ exponential or $p_{\mathrm{T}}$ power law) are likely to reproduce the low-$p_{\mathrm{T}}$ behaviour and are suitable for the low-$p_{\mathrm{T}}$ extrapolation. These functions are fitted to the low-$p_{\mathrm{T}}$ part of the spectrum below $3$ GeV$/c$ and used to evaluate the low-$p_{\mathrm{T}}$ contribution outside the measured range. An $m_{\mathrm {T}}$ exponential functional form

4 $$\begin{aligned} \frac{1}{N_\mathrm{INEL}}\frac{\mathrm {d}^{2}N}{\mathrm {d}y\mathrm {d}p_{\mathrm{T}}}&= A\; p_{\mathrm{T}}\;m_{\mathrm {T}} \;e^{-\frac{m_{\mathrm {T}}}{C}}, \end{aligned}$$

where $A$ is the normalization factor and $C$ is the inverse slope parameter, gives values for d$N$/d$y$ which are $\sim$ 5–6 % lower and values for $\langle p_{\mathrm{T}}\rangle$ which are $\sim$ 3 % higher than those obtained with the Lévy–Tsallis function. A $p_{\mathrm{T}}$ power law functional form

5 $$\begin{aligned} \frac{1}{N_\mathrm{INEL}}\frac{\mathrm {d}^{2}N}{\mathrm {d}y\mathrm {d}p_{\mathrm{T}}}&= A\; p_{\mathrm{T}}\; \left( 1+ \frac{p_{\mathrm{T}}}{nC}\right) ^{-n} , \end{aligned}$$

gives values for d$N$/d$y$ which are $\sim$ 10–15 % higher and values for $\langle p_{\mathrm{T}}\rangle$ which are $\sim$ 9–11 % lower than those obtained with the Lévy–Tsallis function. Arithmetic averages of the values obtained with the three functions (Lévy–Tsallis, $m_{\mathrm {T}}$ exponential, $p_{\mathrm{T}}$ power law) are taken for d$N$/d$y$ and $\langle p_{\mathrm{T}}\rangle$ and the unbiased estimators of standard deviation are considered as systematic uncertainties associated to the low-$p_{\mathrm{T}}$ extrapolation. These systematic uncertainties are summed in quadrature to contribute to the overall systematic uncertainties on d$N$/d$y$ and $\langle p_{\mathrm{T}}\rangle$. Table 8 summaries the results.

**Table 8:** Particle yield per unit rapidity, d$N$/d$y$, and mean transverse momentum, $\langle p_{\mathrm{T}}\rangle$. Values are obtained as an average of the values calculated with three different functions [Lévy–Tsallis (Eq. 3), $m_{\mathrm {T}}$ exponential (Eq. 4), $p_{\mathrm{T}}$ power law (Eq. 5)], which reproduce the low-$p_{\mathrm{T}}$ behaviour of the spectrum. Systematic uncertainties include those from the low-$p_{\mathrm{T}}$ extrapolation and (for d$N$/d$y$ only) the $p_{\mathrm{T}}$-independent uncertainties from Table 6

Baryon	d$N$/d$y$ ($\times$10$^{-3}$)	$\langle p_{\mathrm{T}}\rangle$ (GeV$/c$)
$\Sigma (1385)^{+}$	10.0 $\pm$ 0.2 $^{+1.5} _{-1.4}$	1.15 $\pm$ 0.02 $\pm$ 0.07
$\Sigma (1385)^{-}$	10.8 $\pm$ 0.2 $^{+1.7} _{-1.6}$	1.15 $\pm$ 0.02 $\pm$ 0.08
$\Sigma (1385)^{-}$	9.1 $\pm$ 0.2 $^{+1.5} _{-1.4}$	1.16 $\pm$ 0.02 $\pm$ 0.08
${\overline{\Sigma }}$(1385)$^{+}$	10.3 $\pm$ 0.2 $^{+1.7} _{-1.5}$	1.16 $\pm$ 0.02 $\pm$ 0.07
$\Xi$(1530)$^{0}$	2.56 $\pm$ 0.07 $^{+0.40} _{-0.37}$	1.31 $\pm$ 0.02 $\pm$ 0.09

The anti-baryon to baryon ratios, ${\overline{\Sigma }}$(1385)$^{-}/$ ${\Sigma }$(1385)$^{+}$ and ${\overline{\Sigma }}$(1385)$^{+}/$ ${\Sigma }$(1385)$^{-}$, are compatible with unity, although the large uncertainties leave very little predictive power on the mechanisms of baryon-number transport [33].

### Comparison to models

**Fig. 6:**
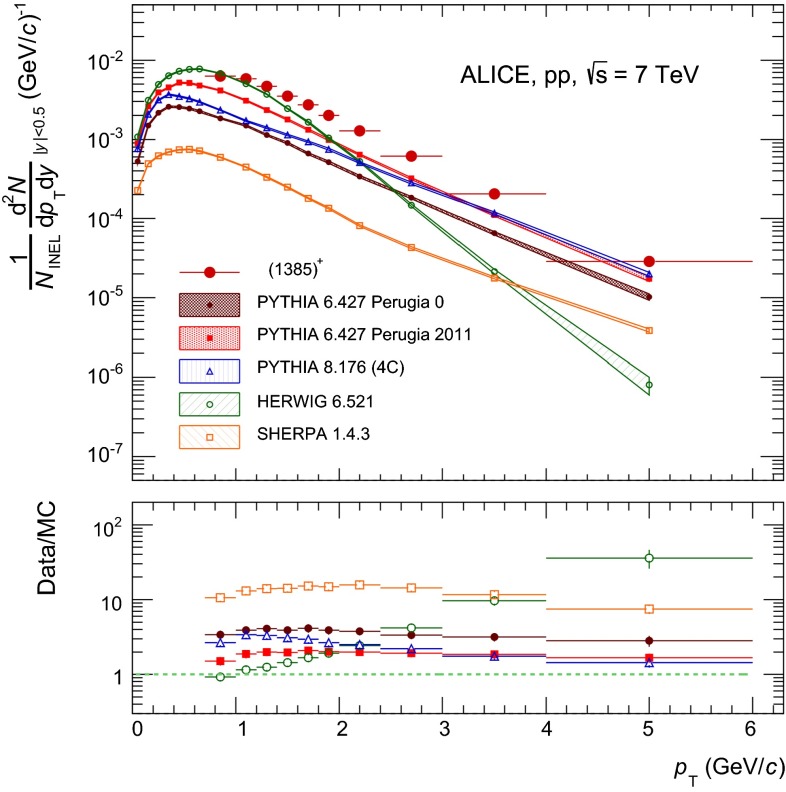
The transverse momentum spectrum of ${\Sigma }$(1385)$^{+}$ is compared to standard tunes of PYTHIA 6 [34] and PYTHIA 8 [35], the latest release of HERWIG (6.521) [36], and SHERPA release 1.4.6 [37]. The MC data are binned according to the data. Spectra points are represented at the centre of the $p_{\mathrm{T}}$ interval. The *lower panel* shows the ratio data/MC. $p_{\mathrm{T}}$-independent uncertainties are not shown

**Fig. 7:**
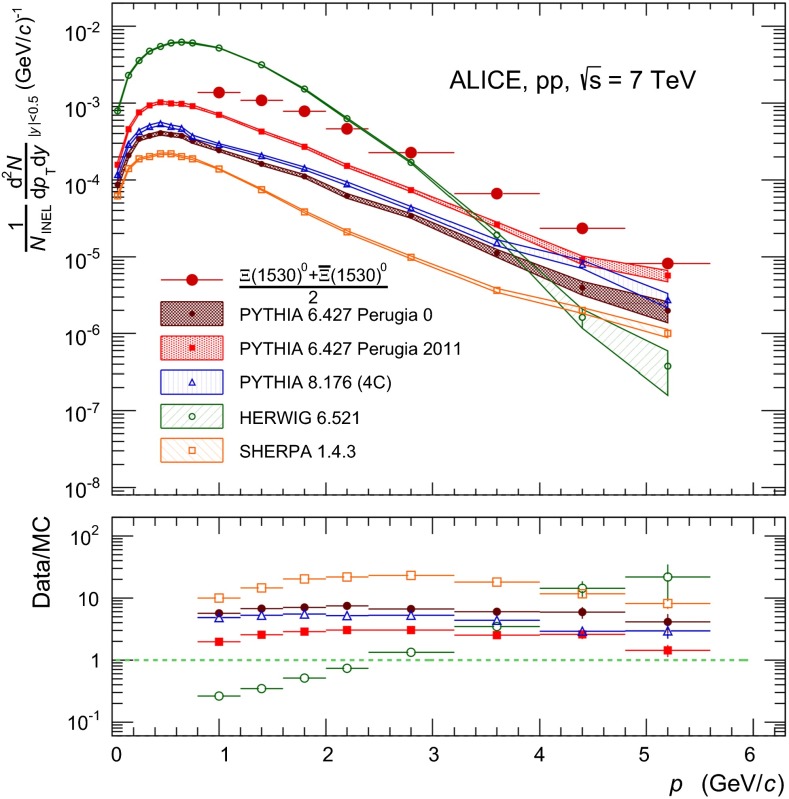
Same as Fig. 6 but for ${\Xi (1530)^{0}}$

The transverse momentum spectra of both ${\Sigma }$(1385) and ${\Xi (1530)^{0}}$ are compared to standard tunes of PYTHIA 6 [34] and PYTHIA 8 [35], HERWIG [36] and SHERPA [37]. This is shown in Figs. 6 and 7 for ${\Sigma }$(1385)$^{+}$ and ${\Xi (1530)^{0}}$, respectively. Similar results to those of ${\Sigma }$(1385)$^+$ are obtained for the other ${\Sigma }$(1385) species.

The latest release of PYTHIA 6 (6.427) is used. One of its latest tunes (Perugia 2011, tune 350 [22]) is compared with the central parameter set (Perugia 0, tune 320). Perugia 2011 takes into account some of the early LHC minimum-bias and underlying-event data at 0.9 and 7 TeV (see [22] and references therein) and describes the 7 TeV pp charged particle spectra reasonably well [30]. The multi-strange baryon yields are also better described by the Perugia 2011 tune, even if it still underpredicts the data [31]. Similar conclusions hold for the strange meson resonances $\phi$ and K$^*$ [20]. For both ${\Sigma }$(1385) and ${\Xi (1530)^{0}}$, the Perugia 2011 tune underestimates the data, though it gives a better description with respect to Perugia 0. Also the Perugia 2012 tune of PYTHIA 6 (tune 370 [38]) has been tested with no significant improvement in the predictions for both ${\Sigma }$(1385) and ${\Xi (1530)^{0}}$. The Perugia 2012 tune [38] is a retune of Perugia 2011 which utilizes a different parton distribution function, CTEQ6L1 instead of CTEQ5L. The predictions from the Perugia 2012 tune are not reported in Figs. 6 and 7.

The latest release of PYTHIA 8 (8.176) is used. The standard 4C tune (CTEQ6L1 [35]) gives a worse description with respect to the Perugia 2011 tune of PYTHIA 6. The 4C tune has color reconnection (CR) enabled by default: switching CR off gives a worse description, as expected [39]. ATLAS tunes A2-MSTW2008LO and AU2-CTEQ6L1 have been considered as alternatives to the standard 4C tune (CTEQ6L1). The A2-MSTW2008LO utilizes a different parton distribution function and the AU2-CTEQ6L1 is better tuned for underlying events. None of them performs better than the 4C tune; therefore, they are not reported in Figs. 6 and 7.

Also shown in Figs. 6 and 7 are the results from HERWIG (release 6.521) [36] and SHERPA (release 1.4.6) [37]. HERWIG predicts a much softer production than for both the other models and the data, for both ${\Sigma }$(1385) and ${\Xi (1530)^{0}}$. For ${\Sigma }$(1385), HERWIG is likely to describe the data at low $p_{\mathrm{T}}$, but it underpredicts the data by a factor $\sim$ 2$-$4 in the intermediate-$p_{\mathrm{T}}$ region $2<$ $p_{\mathrm{T}}$ $<3$ GeV$/c$, and more than one order of magnitude at higher $p_{\mathrm{T}}$. For ${\Xi (1530)^{0}}$, HERWIG fails both at low $p_{\mathrm{T}}$, where the predictions are overestimated by a factor $\sim$ 2$-$4, and at high $p_{\mathrm{T}}$, where the predictions are underestimated by more than one order of magnitude. SHERPA gives a better description of the spectral shape for both ${\Sigma }$(1385) and ${\Xi (1530)^{0}}$, but the overall production cross sections are largely underestimated.

The integrated yields d$N$/d$y$ are also compared to thermal model calculations by Becattini et al. [40], tuned on the yields measured by the ALICE experiment at $\sqrt{s}$ $=$ 7 TeV for $\pi ^{+}$, K$^{*0}$, $\phi$, $\Xi ^{\pm }$ and $\Omega ^{\pm }$ [20, 30, 31], giving a temperature of $T=$ 160 MeV. The other parameters, as obtained from the fit to the ALICE data, are the strangeness suppression factor, $\gamma _{S}$ $=$ 0.72, the normalization parameter, $A$ $=$ 0.0355, and $V T^{3}$ $=$ 231.2, where $V$ is the volume.

The comparison is done for the ratios ${\Sigma }$(1385)$^{+}$/ ${\Lambda }$ and ${\Xi (1530)^{0}}$ $/\Xi ^{-}$, which are sensitive to the temperature $T$. The experimental yields of ${\Lambda }$ and $\Xi ^{-}$ are from [31, 41]. The theoretical value for the ${\Xi (1530)^{0}}$ is obtained as average of the values for ${\Xi (1530)^{0}}$ and ${\overline{\Xi } (1530)^{0}}$, to be compared to the experimental results of this analysis. The theoretical prediction for ${\Sigma }$(1385)$^{+}$ $/$ ${\Lambda }$ (0.13) is in agreement with the measured value (0.131 $\pm$ 0.002 $\pm$ 0.021). Similar conclusions hold for the other ${\Sigma }$(1385) species (namely, for the ratios ${\Sigma }$(1385)$^{-}$ $/$ ${\Lambda }$, ${\overline{\Sigma }}$(1385)$^{-}$ $/$ ${\overline{\Lambda }}$ and ${\overline{\Sigma }}$(1385)$^{+}$ $/$ ${\overline{\Lambda }}$). The prediction for ${\Xi (1530)^{0}}$ $/\Xi ^{-}$ (0.38) is also in agreement with the experimental value ($0.32 \pm 0.01 \pm 0.05$) if both statistical and systematic uncertainties are considered.

### Mean transverse momentum $\langle p_{\mathrm{T}}\rangle$

**Fig. 8:**
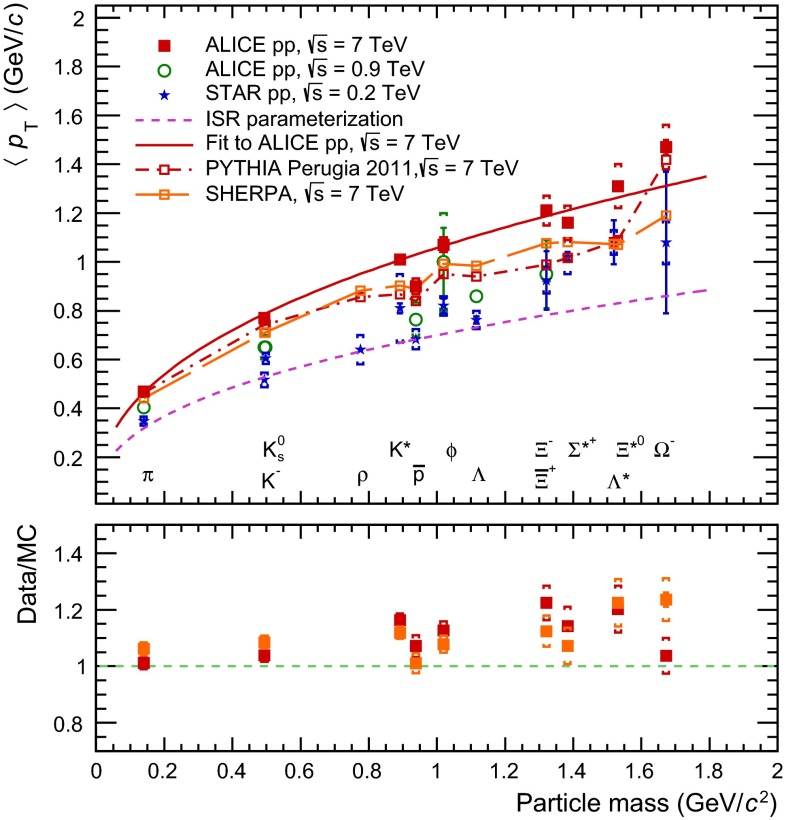
The mean $p_{\mathrm{T}}$ as function of the particle mass including ${\Sigma }$(1385)$^+$, ${\Xi (1530)^{0}}$ and other particles reconstructed in pp collisions at $\sqrt{s}$ $=$ 7 TeV and $\sqrt{s}$ $=$ 0.9 TeV by the ALICE collaboration [19, 20, 30–32] and at $\sqrt{s}$ $=$ 0.2 TeV by the STAR collaboration [1, 42–45]. The *lower panel* shows the ratio data/MC. Statistical and systematic uncertainties are shown separately (*vertical solid lines* and *brackets*, respectively)

The mean transverse momentum $\langle p_{\mathrm{T}}\rangle$ serves as a single variable to characterize the soft part of the measured particle spectra. Figure 8 shows the $\langle p_{\mathrm{T}}\rangle$ as a function of the particle mass, covering a wide range of hadron mass up to the $\Omega ^{-}$.

The plot includes ${\Sigma }$(1385)$^+$ and ${\Xi (1530)^{0}}$ from this analysis, and other particles measured in pp collisions at $\sqrt{s}$ $=$ 0.9 TeV and $\sqrt{s}$ $=$ 7 TeV with the ALICE experiment [19, 20, 30–32]. The STAR pp data at $\sqrt{s}$ $=$ 0.2 TeV [1, 42–45] are added for comparison. The dashed line in Fig. 8 is the ISR parametrization, an empirical curve proposed originally [46] to describe the ISR [47] and FNAL [48] data for $\pi$, K and p only, at $\sqrt{s}$ $=$ 0.025 TeV.

For STAR data, the ISR parametrization still works relatively well for lower-mass particles up to $\sim$ 1 GeV$/c^2$ [44], despite the jump in the collision energy by nearly an order of magnitude with respect to previous experiments, but it fails to describe the dependence of $\langle p_{\mathrm{T}}\rangle$ for higher-mass particles. At the RHIC energies, this was attributed to an increasing contribution to the transverse momentum spectra from mini-jet production [49]. In particular, it was noted that strange baryon resonances ($\Sigma$(1385) and $\Lambda$(1520)) follow a steeper increase, similar to the trend of heavier mass particles [1].

For ALICE data, the ISR parametrization fails to fit the lower-mass particles already at the collision energy of $\sqrt{s}$ $=$ 0.9 TeV and the dependence of $\langle p_{\mathrm{T}}\rangle$ with the mass is even steeper at $\sqrt{s}$ $=$ 7 TeV. Unlike STAR, strange baryon resonances follow the same trend as the lower-mass particles.

At the LHC energies, flow-like effects in pp collisions are investigated [39, 50] which might explain the harder behaviour of transverse momentum spectra, specially for higher mass particles.

The ALICE points at $\sqrt{s}$ $=$ 7 TeV are fitted with a function similar to the ISR parametrization,

6 $$\begin{aligned} \langle p_{\mathrm{T}}\rangle = \alpha \left( \frac{M}{1~\mathrm {GeV}/ {c}^\mathrm{{2}}}\right) ^{\beta }, \end{aligned}$$

where $M$ is the particle mass, obtaining $\alpha$ $=$ (1.06 $\pm$ 0.02) GeV$/c$ and $\beta$ $=$ 0.43 $\pm$ 0.02. For the fit the statistical and systematic uncertainties are summed in quadrature. A $\chi ^{2}$/ndf $=$ 9.61/6 with a probability of 14 %, is obtained. The antiproton $\langle p_{\mathrm{T}}\rangle$ is excluded from the fit since it is off-trend. Including it in the fit changes very little the fit parameters ($\alpha$ $=$ 1.04 GeV$/c$ and $\beta$ $=$ 0.41) but increases the $\chi ^{2}$ ($\chi ^{2}$/ndf $=$ 15.75/7). The values for $\alpha$ and $\beta$ have to be compared with $\alpha _\mathrm{ISR}$ $=$ 0.7 GeV$/c$ and $\beta _\mathrm{ISR}$ $=$ 0.4. The results of the fit are shown with a solid line in Fig. 8.

The dash-dotted line in Fig. 8 is the prediction from PYTHIA 6, tune Perugia 2011. For ${\Sigma }$(1385)$^+$ and ${\Xi (1530)^{0}}$ the MC predictions are $\sim$ 20 % softer than data. The long-dashed line in Fig. 8 is the prediction from SHERPA, which is also softer than data.

## Search for the $\phi (1860)$ pentaquark

**Fig. 9:**
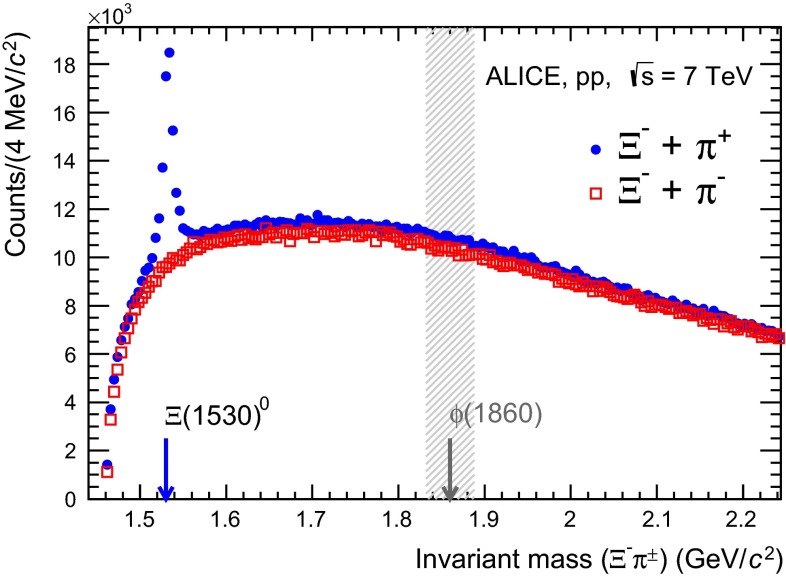
${\Xi }$ $^{-}$ ${\uppi ^+}$ and ${\Xi }$ $^{-}$ ${\uppi ^-}$ invariant mass distributions. The *arrow* and the *shaded area* indicate the region where the $\phi (1860)$ pentaquark is expected and where the search was performed

In order to explore the existence of the $\phi (1860)$ pentaquark, reported by the NA49 experiment [3], the ${\Xi }$ $^{-}$ ${\uppi ^+}$ invariant mass spectrum in Fig. 3 was extended up to above 2 GeV$/c^2$, as shown in Fig. 9.

The arrow and the shaded area give the region where the $\phi (1860)$ pentaquark is expected and where the search was performed. From MC studies with reconstructed particles, the detector mass resolution of the ${\Xi (1530)^{0}}$ is $\sim$ 2 MeV/$c^{\mathrm 2}$ and no significant worsening is expected at masses around 1860 MeV/$c^{\mathrm 2}$. The expected theoretical width of the $\phi (1860)$ is quite narrow ($\lesssim$ 10 MeV/$c^{\mathrm 2}$ [3]) so that, eventually, the detector resolution should not affect the measurement. Also in Fig. 9 the like-sign, ${\Xi }$ $^{-}$ ${\uppi ^-}$, invariant mass distribution is presented. Both channels could potentially exhibit a signature of the $\phi (1860)$ pentaquark: $\phi (1860)^{0}$ in the ${\Xi }$ $^{-}$ ${\uppi ^+}$channel and $\phi (1860)^{-}$ in the ${\Xi }$ $^{-}$ ${\uppi ^-}$channel. Both distributions in Fig. 9 clearly demonstrate the lack of significant evidence for the $\phi (1860)$ pentaquark.

No signal of the pentaquark was found by many other experiments [4–14]. A measure of the maximum likely yield of the $\phi (1860)$ has been made according to the procedure used by the COMPASS experiment [7]. The background is first estimated by fitting the like-sign distribution with a 4$^\mathrm{{th}}$-order polynomial from 1.6 to 2.2 GeV$/c^2$ while excluding the supposed pentaquark range from 1.825 to 1.895 GeV$/c^2$. The signal is counted by integrating the entries in the like-sign distribution in a 28 MeV/$c^{\mathrm 2}$ interval centred around 1.860 GeV$/c^2$. The maximum likely signal estimated at the 3 $\sigma$ ($99\,\%$) confidence level is

7 $$\begin{aligned} S_{\phi (1860)} = 3\sqrt{b} + \mathrm {max}(0,s-b), \end{aligned}$$

where the counted signal and background are given by $s$ and $b$, respectively. The ratio of the integrated ${\Xi }$(1530)$^{0}$ yield to the pentaquark yield, $S_{\phi (1860)}$, is to be compared to other experiments. This is shown in Table 9 for the $\phi (1860)^{-}$. The acceptance effects largely cancel in the ratio. The pentaquark search was also performed moving the centre of the search interval by 10 MeV/$c^{\mathrm 2}$ to the left and to the right; the same result is obtained. Similar results for $S_{\phi (1860)}$ are obtained for $\phi (1860)^{0}$.

**Table 9:** Summary of $\phi$(1860) searches in inclusive production. The energies given in the third column refer to the beam energy in case of fixed-target experiments and to $\sqrt{s}$ in case of collider experiments. The pentaquark signal is related to the ${\Xi (1530)^{0}}$ yield in the last column ${}^\dagger$ At the 95 % CL

Experiment	Initial state	Energy (TeV)	$S_{\phi (1860)^{-}}$	$\Xi$(1530)$^{0}$ /$S_{\phi (1860)^{-}}$
ALICE	pp	$\sqrt{s}=7$	$<$807	$>$44
NA49 [3]	pp	$E_\mathrm{{p}}=0.158$	36	4.2
ALEPH [4]	e$^+$e$^-$	$\sqrt{s}=m_\mathrm{{Z}^{0}}$	$<$24	$>$13.4
BaBar [5]	e$^+$e$^-$	$\sqrt{s}=m_{\Upsilon (4S)}$	not seen	
CDF [6]	p$\bar{\mathrm{p}}$	$\sqrt{s}=1.960$	$<$63	$>$35
COMPASS [7]	$\mu ^+$–A	$E_{{\mu ^{+}}}=0.160$	$<$79	$>$21.5
E690 [8]	pp	$E_\mathrm{{p}}=0.800$	$<$310	$>$302
FOCUS [9]	$\gamma$p	$E_{\gamma } \le 0.300$	$<$170	$>$349
HERA-B [10]	p–A	$E_\mathrm{{p}}=0.920$	$<$56	$>$25
HERMES [11]	e$^-$–D	$E_\mathrm{{e}}=0.0276$	$<$5	$>$7
WA89 [12]	$\Sigma ^-$–A	$E_\mathrm{{\Sigma ^{-}}}=0.340$	$<$760	$>$79
ZEUS [13]	ep	$\sqrt{s}=0.300, 0.318$	not seen	
H1 [14]	ep	$\sqrt{s}=0.300$, 0.318	not seen	$>$2–8${}^\dagger$

## Conclusions

The transverse momentum spectra of the baryon resonances ${\Sigma }$(1385) and ${\Xi (1530)^{0}}$ have been measured by the ALICE collaboration in pp collisions at an energy in the centre of mass of $\sqrt{s}$ $=$ 7 TeV. A Lévy–Tsallis function describes the spectra well.

The mean transverse momentum $\langle p_{\mathrm{T}}\rangle$ of both ${\Sigma }$(1385) and ${\Xi (1530)^{0}}$, when plotted as a function of the particle mass, follows the trend of other particles measured with the ALICE experiment in pp collisions at $\sqrt{s}$ $=$ 7 TeV.

The differential spectra have been compared to several MC event generators, e.g. standard tunes of PYTHIA 6 and PYTHIA 8, HERWIG and SHERPA. PYTHIA 6 Perugia 2011 (tune 350) performs better than any other tested generator, still underpredicting the data by a factor $\sim$ 2-3 in the intermediate-$p_{\mathrm{T}}$ region $2<$ $p_{\mathrm{T}}$ $<3$ GeV$/c$.

The search for the $\phi (1860)^{0}$ and $\phi (1860)^{-}$ pentaquark states in the $\Xi \pi$ charged channels has shown no evidence for the existence of such exotic particles.

## Footnotes

1 BR $\sim$ 100 % for $\Xi (1530)\rightarrow \Xi \pi$ [17], then BR $=$ ($\frac{1}{3}\times$100) % for $\Xi (1530)^{-}\rightarrow \Xi ^{-}\pi ^{0}$ and BR $=$ ($\frac{2}{3}\times 100$) % for $\Xi (1530)^{-}\rightarrow \Xi ^{0}\pi ^{-}$ due to isospin considerations.

2 BR $=$ ($11.7 \pm 1.5$) % [17] for $\Sigma (1385)\rightarrow \Sigma \pi$, then BR $=$ ($\frac{1}{2}\times$11.7) % for the charged-pion channel $\Sigma (1385)^{\pm }\rightarrow \Sigma ^{0}\pi ^{\pm }$ due to isospin considerations.

3 BR $=$ $(10 \pm 1)\,\%$ [17] for $\Lambda (1520)\rightarrow \Lambda \pi \pi$, then BR $=$ ($\frac{1}{2}\times$10) % for the charged-pions channel $\Lambda (1520)\rightarrow \Lambda \pi ^{\pm }\pi ^{\mp }$ due to isospin considerations.
